# Efficacy and Safety of Isotonic and Hypotonic Intravenous Maintenance Fluids in Hospitalised Children: A Systematic Review and Meta-Analysis of Randomised Controlled Trials

**DOI:** 10.3390/children8090785

**Published:** 2021-09-08

**Authors:** Norfarahin Hasim, Mimi Azliha Abu Bakar, Md Asiful Islam

**Affiliations:** 1 Department of Emergency Medicine, School of Medical Sciences, Universiti Sains Malaysia, Kubang Kerian 16150, Kelantan, Malaysia; farahinhashim@student.usm.my; 2 Hospital Universiti Sains Malaysia, Kubang Kerian 16150, Kelantan, Malaysia; 3 Department of Haematology, School of Medical Sciences, Universiti Sains Malaysia, Kubang Kerian 16150, Kelantan, Malaysia

**Keywords:** isotonic, hypotonic, intravenous fluid, efficacy, safety, hospitalised, children, systematic review, meta-analysis

## Abstract

Hyponatraemia is a known complication in hospitalised children receiving maintenance intravenous fluid. Several studies have been published to investigate the efficacy and safety of intravenous fluids in children. However, there is still an ongoing debate regarding the ideal solution to be used in the paediatric population. Therefore, the aim of this meta-analysis was to investigate the safety and efficacy of administering isotonic versus hypotonic intravenous maintenance fluid in hospitalised children. An extensive search was undertaken on PubMed, Web of Science, Scopus, ScienceDirect, Google Scholar and Cochrane Library on 28 December 2020. Only randomised controlled trials (RCTs) were included. We used the random-effects model for all analyses. Risk ratio (RR) and mean difference with 95% confidence intervals (CIs) were used for dichotomous and continuous outcomes, respectively. The quality of each study was assessed using the Joanna Briggs Institute critical appraisal tool for RCTs. This study is registered with PROSPERO (CRD42021229067). Twenty-two RCTs with a total of 3795 participants were included. The studies encompassed surgical and medical patients admitted to intensive care unit as well as to general wards. We found that hypotonic fluid significantly increases the risk of hyponatremia at both ≤24 h (RR 0.34; 95% CI: 0.26–0.43, *p* < 0.00001) and >24 h (RR 0.48; 95% CI: 0.36–0.64, *p* < 0.00001). Isotonic fluid increases the risk of hypernatraemia at ≤24 h (RR 2.15; 95% CI: 1.24–3.73, *p* = 0.006). The prevalence of hyponatraemia was also higher in the hypotonic group at both ≤24 h (5.7% vs. 23.3%) and >24 h (6.0% vs. 26.3%). There was no statistically significant difference in the risk of developing adverse outcomes between the two groups. Mean serum and urine sodium as well as serum osmolality/osmolarity was lower in the hypotonic group. Isotonic solution is protective against the development of hyponatraemia while hypotonic solution increases the risk of hyponatraemia.

## 1. Introduction

The practice of prescribing hypotonic solution as maintenance intravenous fluid (IVF) in children was made popular more than six decades ago following the recommendation proposed by Holliday and Segar in 1957 [1]. Their recommendations were derived based on the caloric expenditure of healthy children as well as the electrolyte composition of human and cow’s milk [1]. These days, this equates to a 0.2% sodium chloride in a 5% dextrose solution [2], which is markedly hypotonic in comparison to plasma tonicity. This practice has since been called into question. Moritz and Ayus [3], in their 2003 review, highlighted the dangers of prescribing hypotonic fluid in children. They reported over 50 deaths and significant undesirable neurological outcomes linked to hypotonic fluid; hence, they proposed that an isotonic solution would be a better choice as maintenance IVF in children.

The goal of initiating maintenance IVF is to help maintain a normal electrolyte balance when oral intake is insufficient to preserve the extracellular volume [4,5,6]. A wide range of fluids is commercially available. They can all be classified as either isotonic or hypotonic solutions. The osmolality of an isotonic solution is equal to or close to that of plasma (275–295 mOsm/kg), whereas the osmolality of a hypotonic solution is lower than that of plasma [7]. The constituents of these fluids differ from one another [8,9,10]. The variety of available fluids, combined with a lack of knowledge about their components, can make it difficult for physicians to choose the best solution for their patients [11,12,13].

The main concerns of using hypotonic solution are the development of hyponatraemia and its neurological effects. Hyponatraemia in the hospitalised paediatric population results from two factors. The first is the administration of electrolyte-free water such as hypotonic saline and the second is the secretion of antidiuretic hormone (ADH) from the posterior pituitary gland, which prevents the excretion of this electrolyte-free water [14]. This effect of the hypotonic solution makes it less desirable as IVF in children. Isotonic fluid does not affect plasma osmolality since it contains sodium at physiological plasma concentrations [15]. The infused isotonic fluid will be distributed freely within the extracellular fluid (ECF) compartment causing a minimal change in the sodium concentration and osmolality [16], thereby, limiting the movement of water from the ECF into the intracellular fluid (ICF) compartment and vice versa. This lack of shift of water between the ECF and ICF is critical in preventing cerebral oedema caused by hyponatraemia which can lead to significant neurological morbidity.

Little attention has been placed on urine chemistries; however, they are important in the workup for dysnatraemia [17,18,19]. There are no “normal values” for urine electrolyte concentrations, but there are “expected values” [17,18]. The findings of these figures must be interpreted in light of the clinical context. For example, in a dehydrated patient, urine sodium is anticipated to be low as there will be water conservation. When dealing with dysnatraemia, it may be possible to determine the cause of the electrolyte imbalance by combining the results of plasma and urine osmolality with urine sodium as well as clinical examination [18,20,21].

The American Academy of Pediatrics (AAP) published a guideline in 2018 recommending isotonic solutions as intravenous maintenance fluid therapy in children aged from 28 days to 18 years old [5]. The National Institute for Health and Care Excellence (NICE) issued an updated guideline in 2020 that suggested the use of isotonic fluid in term neonates over eight days old [22]. Since the publication of these guidelines, there has been a shift towards prescribing isotonic solutions [23,24] among physicians compared to previous years [25,26,27,28,29]. Several interventional studies have been carried out to improve adherence to the recommendation by AAP [30,31]. These studies reveal that although guidelines are available, there are still physicians who prescribe hypotonic fluid as IVF in children.

Despite mounting evidence of the dangers of using hypotonic IVF, many physicians continue to recommend it. There may exist doubts regarding the safety of isotonic fluid due to concerns that it might induce hypernatraemia and fluid overload [32,33] in children. Therefore, we intend to investigate the efficacy and safety of isotonic versus hypotonic solutions as maintenance IVF in hospitalised children by conducting a systematic review and meta-analysis. We also aimed to analyse the occurrence of adverse events associated with IVF.

## 2. Methods

### 2.1. Search Strategy

In accordance with the PRISMA guidelines [34], we conducted a systematic review and meta-analysis of the literature to identify studies that compared the effects of isotonic versus hypotonic maintenance fluid in hospitalised children. This study is registered with the PROSPERO database (registration number: CRD42021229067). Initial searches were not restricted by date, language, or study design. PubMed, Web of Science, Scopus, Google Scholar, and Cochrane Library databases were searched on 28 December 2020. The following keywords were searched: isotonic, hypotonic, saline, hyponatremia, hyponatraemia, children, pediatric, paediatric, pediatrics, paediatrics, adolescent, adolescents, child, infant, infants, newborn, and newborns. Complete detail of search strategies is in the Supplementary Table S1. The listed studies’ references were also reviewed to ensure a thorough search. Using the EndNote X8 programme, we were able to remove duplicate studies.

### 2.2. Eligibility Criteria

The population considered were hospitalised patients aged newborn till 18 years old with any medical or surgical conditions requiring maintenance IVF. Our aim was to analyse isotonic versus hypotonic fluid as maintenance IVF in hospitalised children. The primary outcome was hyponatraemia (defined as serum sodium ≤ 135 mmol/L). Secondary outcomes were hypernatraemia (defined as serum sodium ≥ 145 mmol/L), change in serum and urine sodium levels following infusion of fluid, serum osmolality/osmolarity, and adverse outcomes observed during the study. Hypotonic fluid is defined as any fluid which has tonicity lower than that of 0.9% sodium chloride, such as 0.45% sodium chloride or 0.18% sodium chloride. An isotonic fluid is defined as normal saline (0.9%), Hartmann’s solution, Ringer’s lactate, and any other fluid with osmolality close to that of plasma [35]. Other inclusion criteria were as follows: randomised controlled trials and studies comparing isotonic with hypotonic fluid. The following were excluded: review articles, conference abstracts, animal studies, opinions and perspectives, non-randomised controlled trials, and case reports. Data gathered from databases, websites or reported in press releases and news reports were not considered. Additionally excluded were studies that involved patients with dysnatraemia before starting the study that is defined as serum sodium ≤ 130 or ≥145 mmol/L.

### 2.3. Study Selection

The titles and abstracts of articles of interest were first screened to identify eligible studies. Following that, the full texts of the aforementioned articles were evaluated. Disagreements regarding including a study were resolved after discussion among authors.

### 2.4. Data Extraction

One author (N.H.) extracted the data of interest, and other authors (M.A.I. and M.A.A.B.) cross-checked the data independently and disagreements were resolved by discussions. From each of the eligible studies, the following information were entered into an Excel spreadsheet: first author’s last name, year and country of the study, duration of follow-up, total number of participants, characteristic of the study population (number, age, and surgical or medical condition), description of interventions and comparisons as well as outcomes.

### 2.5. Subgroup and Sensitivity Analyses

All the primary and secondary outcomes except adverse events were subgrouped based on the duration of the fluid interventions (i.e., ≤24 and >24 h). Whenever there were multiple outcome measurement times (i.e., 8, 12, 18, 36, 48, and 72 h), the data at the longest fluid administration time was selected for analysis and to avoid duplication of data. In addition, we conducted another subgroup analysis based on different concentrations of the maintenance fluids (i.e., 0.9% vs. 0.45% and 0.9% vs. 0.18%). All sensitivity analyses were carried out with data taken at 24 h. This time point was chosen as most studies had outcome measurements at 24 h. We performed sensitivity analyses for the risks of hyponatraemia and hypernatraemia, as well as the mean difference in serum and urine sodium after excluding small studies with less than 100 participants, low- or medium-quality studies, and if we were to conduct the analysis using a fixed-effects model [36,37]. We also performed an additional sensitivity analysis excluding studies that had changes in the types of maintenance fluid during the study period for risks of hyponatraemia and hypernatraemia.

### 2.6. Quality Assessment and Publication Bias

Two authors (N.H. and M.A.A.B.) independently evaluated the quality of included studies using the Joanna Briggs Institute critical appraisal tool for randomised controlled trials [38]. If the total score was <50%, 50–70%, or >70%, the studies were categorised as low quality (high risk of bias), moderate quality (moderate risk of bias), and high quality (low risk of bias) [37,39]. In main and subgroup analysis, if there was a minimum of 10 studies, publication bias was analysed and visually represented assessing the primary outcomes (i.e., hyponatraemia and hypernatraemia).

### 2.7. Data Analysis

Risk ratio (RR) and mean difference (MD) with 95% confidence intervals (CIs) were used for dichotomous and continuous outcomes, respectively. Additionally, pooled prevalence was estimated with 95% CI. All the analyses were calculated using the random-effects model. We employed the Mantel-Haenzel method to estimate dichotomous outcomes and the inverse variance analysis method for the continuous outcomes. The *I*^2^ statistic was used to assess heterogeneity (*I*^2^ > 75% indicating significant heterogeneity), as well as Cochran’s Q-test to establish the significance of heterogeneity. RevMan (version 5.4) was used to create all of the analyses and plots [40] and metaprop codes in meta (version 4.15-1) and metafor (version 2.4-0) packages of R (version 3.6.3) in RStudio (version 1.3.1093) software (RStudio, Inc., Boston, MA, USA) [23].

## 3. Results

### 3.1. Study Selection

**Figure 1:**
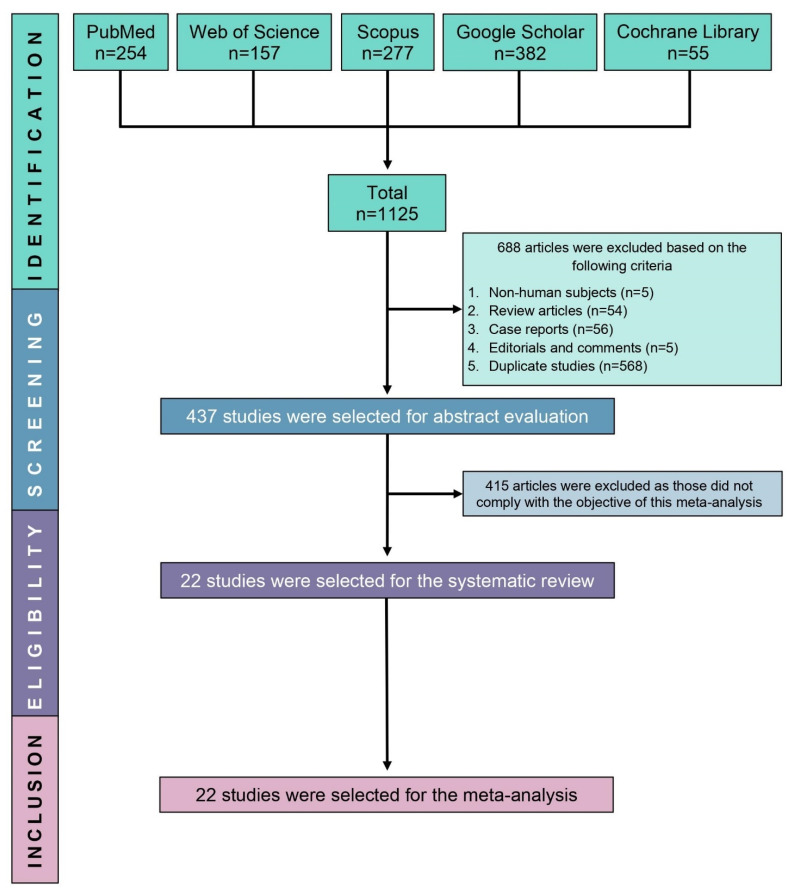
PRISMA flow diagram of study selection.

Our initial search identified 1125 studies. We eliminated 688 articles for the following reasons: non-human subjects (*n* = 5); review articles (*n* = 54); case reports (*n* = 56); editorials and comments (*n* = 5); duplicate studies (*n* = 568). In total, 437 studies were screened for eligibility, from which 415 were removed as they did not meet the objective of the meta-analysis. The full texts of the remaining 22 studies were reviewed, and finally, all these 22 studies were included (Figure 1).

### 3.2. Study Characteristics

Table 1 summarises the major characteristics of the included studies. This meta-analysis is based on a study of 3795 patients hospitalised for various surgical and medical conditions. The studies were conducted in nine different countries: India (*n* = 8), Australia (*n* = 3), Canada (*n* = 4), Argentina (*n* = 2), Portugal (*n* = 1), Finland (*n* = 1), Poland (*n* = 1), Spain (*n* = 1), and Nigeria (*n* = 1). Sixteen (72.7%) of the studies used 0.9% sodium chloride as the isotonic solution. Ten (45.5%) of the studies used 0.45% sodium chloride as the hypotonic solution. The duration of fluid therapy and the timing of outcome measurements varied between studies, ranging from less than 8 h to seven days after the start of the maintenance IVF.

### 3.3. Primary Outcomes

**Figure 2:**
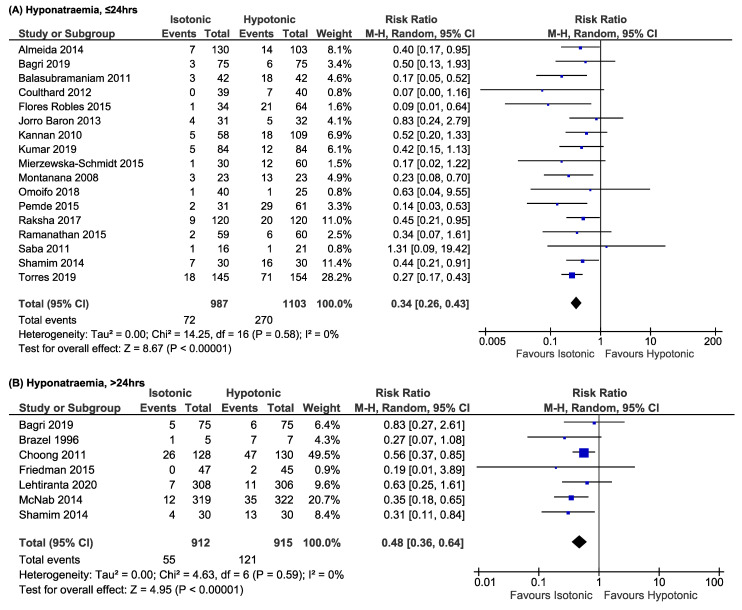

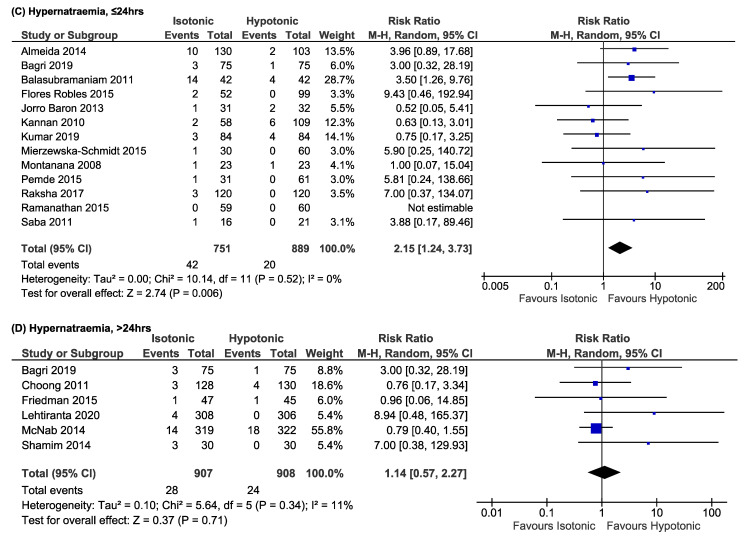
Risks of hyponatraemia (**A**,**B**) and hypernatraemia (**C**,**D**) following isotonic versus hypotonic fluid at ≤24 and >24 h.

**Figure 3:**
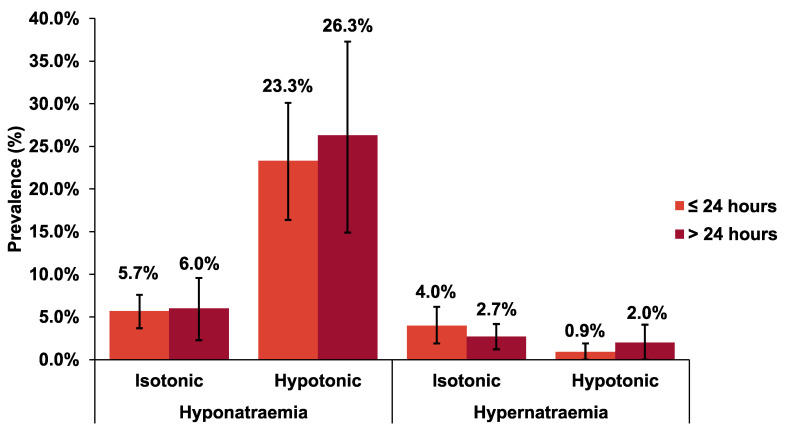
Prevalence with 95% CIs of hyponatraemia and hypernatraemia following isotonic and hypotonic fluids in hospitalised children at ≤24 and >24 h.

Our subgroup analysis at 8, 12, 18, 24, 36, 48, and 72 h showed that isotonic fluid reduced the risk of hyponatraemia where the lowest relative risks were observed at 18 h (RR 0.21; 95% CI: 0.07–0.59) and 36 h (RR 0.21; 95% CI: 0.07–0.67) (Figure S1). Figure 2, Figure 3 and Figure S2 summarise the analyses on risks and prevalence of hyponatraemia following infusion of isotonic and hypotonic fluids. Isotonic fluid significantly decreased the risks of hyponatraemia at both ≤24 h (RR 0.34; 95% CI: 0.26–0.43; *p* < 0.00001; *I*^2^ = 0%) and >24 h (RR 0.48; 95% CI: 0.36–0.64; *p* < 0.00001; *I*^2^ = 0%) (Figure 2). We found that the prevalence of hyponatraemia in the isotonic group is lower compared to the hypotonic group at both ≤24 and >24 h; 5.7% (95% CI: 3.7–7.6) and 6.0% (95% CI: 2.3–9.6), respectively (Figure 3 and Figure S2). Analysis on 0.9% vs. 0.18% sodium chloride and 0.9% vs. 0.45% sodium chloride revealed comparable relative risks in the development of hyponatraemia; RR 0.40 (95% CI: 0.26–0.60; *p* < 0.0001; *I*^2^ = 0%) and RR 0.31 (95% CI: 0.22–0.44; *p* < 0.00001; *I*^2^ = 0%), respectively (Figure S3).

### 3.4. Secondary Outcomes

Hypernatraemia was evaluated in 78.3% of our included studies. Isotonic fluid was shown to increase the risk of hypernatraemia at ≤24 h (RR 2.15; 95% CI: 1.24–3.73; *p* = 0.006; *I*^2^ = 0%) (Figure 2). However, after >24 h of IVF infusion, there was no statistically different risk of hypernatraemia when using isotonic or hypotonic fluid (RR 1.14; 95% CI: 0.57–2.27; *p* = 0.71; *I*^2^ = 11%) (Figure 2). Prevalence of hypernatraemia is higher in the isotonic group at both outcome measurement times: 4.0% (95% CI: 1.9–6.2, *p* = <0.01; *I*^2^ = 58%) and 2.7% (95% CI: 1.2–4.2, *p* = 0.14; *I*^2^ = 39%) (Figure 3 and Figure S2).

Mean serum sodium level was lower in those receiving hypotonic fluid (Figure S4). At ≤24 h, there was a statistically significant decrease of 2.50 mEq/L (95% CI: 1.53–3.46, *p* = <0.00001; *I*^2^ = 90%). In contrast, after 24 h, the decrease was not statistically significant; 2.05 mEql/L (95% CI: −2.00–6.11, *p* = 0.32; *I*^2^ = 96%).

Data on urine sodium were available for 1295 participants from five studies (28–32) (Figure S4). The analyses showed a statistically significant decrease in urine sodium in the hypotonic group with wide 95% CIs and substantial heterogeneity. There was a decrease of 45.05 mmol/L (95% CI: 21.70–68.39; *p* = 0.0002; *I*^2^ = 91%) at ≤24 h and 51.36 mmol/L (95% CI: 24.73–77.99; *p* = 0.0002; *I*^2^ = 89%) at > 24 h in the hypotonic group.

Since the difference in serum osmolarity and osmolality in humans is negligible (33), we pooled the results into the analysis. At ≤24 and >24 h, serum osmolarity and osmolality were lower in the hypotonic group; 9.80 (95% CI: 3.12–16.48; *p* = 0.004; *I*^2^ = 96%) and 11.76 (95% CI: −1.57–25.09; *p* = 0.08; *I*^2^ = 97%), respectively (Figure S4).

**Figure 4:**
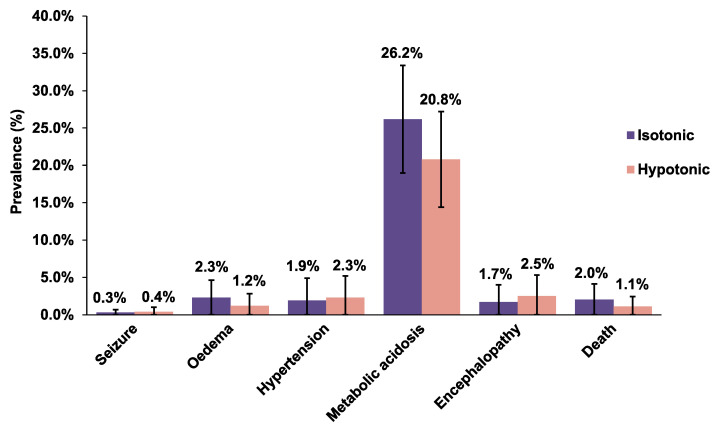
Prevalence with 95% CIs of adverse events following isotonic and hypotonic fluids in hospitalised children.

Some adverse outcomes observed in the included studies were the incidence of seizure, oedema, hypertension, metabolic acidosis, encephalopathy, and death (Figure 4 and Figures S5 and S6). There is little evidence to say for sure that one fluid is more likely to cause serious harm compared to the other one as none of the results were statistically significant. The following are the outcomes of our analyses (Figure S6) for the risks of the development of adverse outcomes: seizure (RR 0.45; 95% CI: 0.08–2.67), oedema (RR 1.41, 95% CI: 0.81–2.46), hypertension (RR 0.90; 95% CI: 0.42–1.93), metabolic acidosis (RR 1.26; 95% CI: 0.84–1.90), encephalopathy (RR 0.67, 95% CI: 0.11–3.92), and death (RR 1.48; 95% CI: 0.72–3.06).

Figure 4 and Figure S5 outline the prevalence of each adverse outcome. The prevalence of seizure is similar in both isotonic and hypotonic groups, 0.3% (95% CI: 0.0–0.7) and 0.4% (95% CI: 0.0–1.0), respectively. Oedema occurred in a total of 29 out of 724 patients in the isotonic group (2.3%; 95% CI: 0.0–4.6) whereas 20 out of 771 patients developed oedema in the hypotonic group (1.2%; 95% CI: 0.0–2.8). The prevalence of hypertension was lower in the isotonic group (1.9%; 95% CI: 0.0–4.9) compared to the hypotonic group (2.3%; 95% CI: 0.0–5.2). As for the prevalence of metabolic acidosis, it was almost similar in both isotonic and hypotonic groups; 26.2% (95% CI: 19–33.4) versus 20.8% (95% CI: 14.4–27.2). Encephalopathy was seen in 1.7% (95% CI: 0.0–4.0) of the participants in the isotonic group, while 2.5% (95% CI: 0.0–5.3) of the participants had encephalopathy in the hypotonic group. The prevalence of death was higher in the isotonic group, 2.0% (95% CI: 0.0–4.1) compared to 1.1% (95% CI: 0.0–2.4) in the hypotonic group.

A summary of the sensitivity analyses can be found in Table S2, where we observed that by excluding small studies (*n* < 100), excluding low- or medium-quality studies, using a fixed-effects model or excluding studies with a change of maintenance fluid did not change any of the results (hyponatremia, hypernatraemia, serum sodium levels or urine sodium levels at 24 h) remarkably.

### 3.5. Quality Assessment and Publication Bias

**Figure 5:**
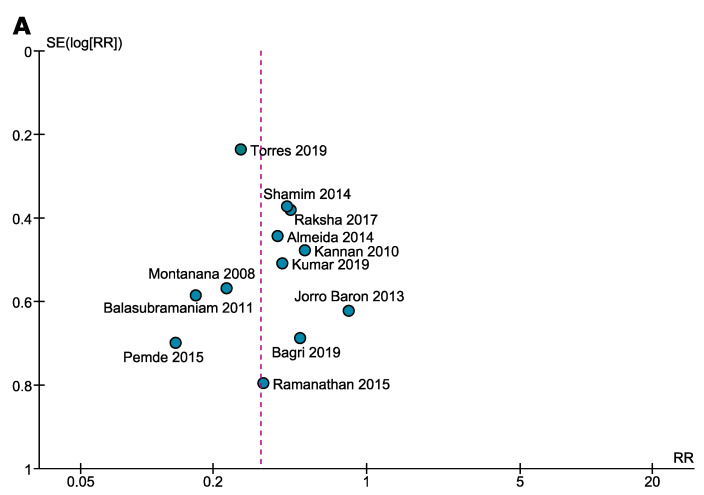

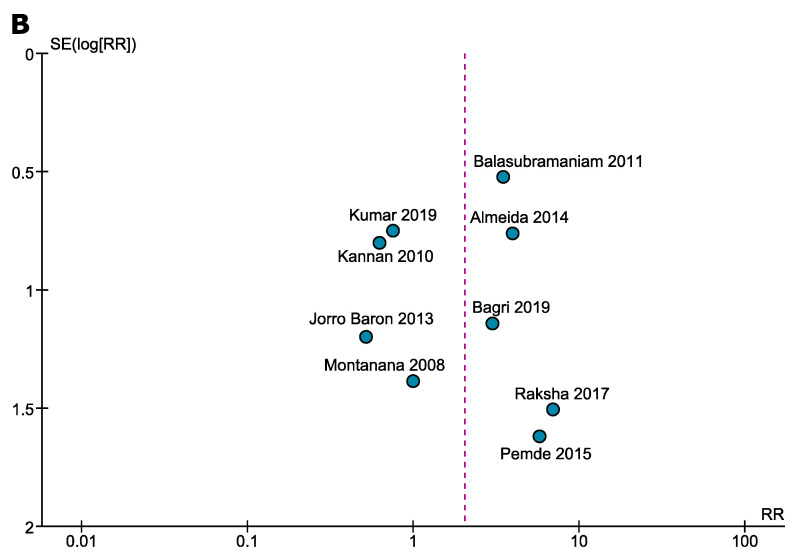
Funnel plots visually representing publication bias assessing risk ratio of (**A**) hyponatraemia and (**B**) hypernatraemia.

Table S3 shows the quality assessment of all 22 studies. Based on our assessment, 77.3% (*n* = 17) of the included studies were of high quality (low risk of bias) while the rest, 22.7% (*n* = 5) were of moderate quality (moderate risk of bias). None of the included studies were classified as low quality (high risk of bias). Visual representation of the funnel plots along with the Egger’s tests depicted the existence of significant publication bias (Figure 5 and Figure S7).

## 4. Discussion

The outcomes of our meta-analysis revealed that isotonic fluid protects children receiving IVF from developing hyponatraemia. Isotonic solutions do not theoretically expand the ICF compartment, thus, preserving the cellular structure and integrity [62]. This is especially important in the paediatric population as they have a higher brain to intracranial volume ratio [2], rendering them more vulnerable to developing complications such as cerebral oedema compared to adults. Therefore, physicians must exercise extreme caution when prescribing IVF in children to avoid unintended complications.

Hospital-acquired hyponatraemia is entirely preventable. Although in our included studies, an assortment of hypotonic fluids was used, the outcome of each included study was comparable indicating that any type of hypotonic fluid, i.e., 0.45% or 0.18% will increase the risk of iatrogenic hyponatraemia. We found no evidence that one hypotonic solution is more likely than the other to cause hyponatraemia (Figure S3), hence any type of hypotonic fluid should generally be avoided. We also discovered that isotonic fluid is consistently protective against hyponatraemia at 8, 12, 18, 24, 36, 48, and 72 h. This means that isotonic fluid is a safer choice till at least 72 h of treatment. Further research is needed to study the effect of fluid on sodium balance in children when it is used for longer duration.

Holliday et al. [63] proposed that isotonic solution increases sodium load when its use is prolonged. Our findings, however, do not support this hypothesis. This is in line with previous studies [64,65,66,67,68,69]. Our data demonstrated that after 24 h, there is no significant difference in the risk of developing hypernatraemia. Thus, there should be no reservations in using isotonic solutions as maintenance fluid. However, a child may not require parenteral fluid supplementation for a prolonged period. The duration of fluid therapy depends on the reason for admission [70]. This makes studying the long-term effect of IVF on the occurrence of hypernatraemia challenging. Nevertheless, this should never take away a physician’s clinical judgment. Fluid supplementation should always be adjusted to the needs of the child and assessed regularly, particularly in critically ill children [71]. This is the only means to ensure that unfavourable events are avoided.

In our investigation, the hypotonic group had lower mean serum and urinary sodium, as well as lower serum osmolarity/osmolality. As the fluid is provided for a longer period, the mean difference in sodium level lessens, which makes sense because a child likely to improve with therapy, resulting in fewer non-osmotic ADH stimuli and hence less free-water retention. It is unclear if the duration of fluid therapy will directly affect the mean sodium level, i.e., longer fluid therapy will result in lower serum sodium level, but we can conclude that hypotonic fluid will cause a fall in sodium levels. This is especially important in patients suffering from chronic diseases like cirrhosis or nephrotic syndrome where hypervolemic hyponatraemia is likely already present. Few studies looked at urine sodium and serum osmolarity/osmolality, making inferences on the impact of solution types on these variables challenging. Nonetheless, our findings indicated that hypotonic fluid lowers serum osmolarity/osmolality, which is understandable given that it provides electrolyte-free water.

The persistent non-osmotic ADH stimuli that a child experiences may explain why hyponatraemia persists even after 72 h of treatment (Figure S1). Hyponatraemia results from the kidney’s inability to excrete free-water or excessive water intake [72]. This is influenced by the thirst mechanism which is activated as plasma osmolality increases. However, in hospitalised individuals, euvolemic hyponatraemia usually occurs because of sustained ADH release in the absence of appropriate osmotic stimulus such as raised plasma osmolality [73].

ADH plays an immense role in sodium homeostasis as described previously. It can promote hyponatraemia by increasing the permeability of collecting ducts in the nephron, consequently causing the retention of free water. Hospitalised children are more likely to develop hyponatraemia, which inhibits their ability to excrete free-water due to numerous non-osmotic ADH secretion triggers such as pain, stress, dehydration, and post-operative effects [74]. Furthermore, when maintenance fluid is prescribed, the IVF supplementation is determined by the physician, not the patients. Not many studies have included ADH measurement as an outcome. Choong et al. [45], Coulthard et al. [46], and Kannan et al. [40] reported serum ADH levels after infusion of IVF. However, the data was not normally distributed; hence, we could not carry out additional analysis. The serum ADH levels in all three studies were elevated, but the results did not differ significantly between the treatment arms. As neither the physician nor the patient can control ADH secretion, all fluid prescribers must be aware of the impact ADH secretion has on a patient’s fluid balance.

Despite the fact that patients receiving isotonic fluid have about 50% higher mortality rate during their stay, a comprehensive evaluation of the causes of mortality found that they had nothing to do with sodium levels. Kannan et al. [40] reported one death (1.7%) in the isotonic group. The cause of death was acute respiratory distress syndrome, and the patient was normonatraemic throughout the study period. On the other hand, Jorro-Baron et al. [49] found three deaths (9.4%) in the hypotonic group. The cause of death was unrelated to the maintenance fluid infusion. All three patients had serum sodium above 130 mmol/L throughout the study. Only one study, Ramanathan et al. [57], reported death (*n* = 2) associated with severe hyponatraemia (serum sodium < 125 mmol/L) in the hypotonic group. However, the causes of death were attributed to respiratory failure. Bagri et al. [42] and Raksha et al. [58] reported deaths in both treatment arms. The patients were nomonatraemic in Rasksha’s study.

From our investigation, we observed that none of the fluids increased the risks of adverse events significantly. Most studies had safety restrictions integrated into their methodology to safeguard against serious adverse outcomes. For instance, in Choong et al. [45], the initial methodology was fully blinded; however, during the study, 24 out of 258 patients (9.3%) were modified to open-label maintenance fluid. The most common reason for the change was hyponatraemia. Although these safety measures may not reveal the actual effects of isotonic versus hypotonic fluid in the development of adverse outcomes, it is unethical to continue administering the fluid to patients or to refuse to intervene when it is apparent that it is causing harm. Moreover, only 15 (68.2%) of the included studies reported adverse outcomes and less than 10 studies have data available for each adverse outcome we were looking at. With this small number of reported data, this potentially does not depict the true risks and prevalence of adverse outcome associated with IVF.

While our analysis showed that hyponatraemia does not directly cause mortality or morbidity, it has been well documented that hyponatraemia is strongly associated with increased risks of death and adverse events [75,76,77]. This causal relationship has also been exhibited in the adult population [78]. Since our research has demonstrated that hypotonic fluid is related to hyponatraemia, it is advisable to avoid using hypotonic fluid as maintenance IVF. Still, we encourage physicians to carefully monitor and observe their patients during IVF therapy [79].

Another criticism against the use of isotonic solutions is the development of metabolic acidosis attributed to the excess chloride administration. One study specifically looking into this outcome was included in our analysis. In a study conducted by Torres et al. [61], there were no significant differences in the incidence of metabolic acidosis between the two treatment arms (Figure 4 and Figure S5). Other studies have found that isotonic fluid causes metabolic acidosis, but many of them involved rapid fluid infusion [80,81,82,83,84,85]. In a retrospective cohort study conducted by Bulfon et al., they have found that when 0.9% sodium chloride is used as both bolus and maintenance fluid, there is an increased risk of developing hyperchloraemic metabolic acidosis (HCMA). However, when used only as maintenance fluid, 0.9% sodium chloride was not an independent risk factor for the occurrence of HCMA [86]. A study examining the effect of isotonic solution as a maintenance IVF on the development of metabolic acidosis, specifically in children, is needed to validate this point.

We performed a thorough search strategy in accordance with the PRISMA guideline and excluded studies in which patients had hyponatraemia prior to the infusion of the study fluid. We also looked at the risks of hyponatraemia at various time points, which revealed that hyponatraemia can occur as early as 6 h when hypotonic fluid is used and can last up to at least 72 h. Apart from that, we performed a subgroup analysis looking at data on 0.9% versus 0.45% and 0.9% versus 0.18% sodium chloride at 24 h (Figure S3). Furthermore, none of the research included was of poor quality. To the best of our knowledge, this is the only meta-analysis that examined the risks and prevalence of hypo and hypernatraemia at both ≤24 and >24 h in hospitalised children. In addition, from our sensitivity analysis, we found that results did not change remarkably, representing the robustness and reliability of our findings.

Only published studies were considered. A variety of fluids were also used in the included studies. This could explain the significant heterogeneity observed in the analysis of the continuous data. Our research included both surgical and medical patients. As these two groups of patients are likely to be exposed to different non-osmotic ADH triggers, the risk of hyponatraemia may not be the same in all hospitalised children. We could not investigate the impact of infusion rate because there was insufficient data. One study, however, found that fluid restriction does not reduce the risk of hyponatraemia [87]. One of the studies included [40] compared isotonic fluid to hypotonic fluid at maintenance rates and hypotonic fluid at limited rates in a three-arm trial. Three other papers [47,53,56] compared isotonic fluid with two types of hypotonic fluids. We combined the data from the hypotonic treatment arm in our analysis. As the fluids’ compositions are dissimilar, this may introduce bias. We also included one study conducted on neonates [43], however, as their renal handing of fluid and electrolytes are likely to be different to the other population in our study [88,89], our analysis may not be applicable to this age group. Nonetheless, other published studies have also shown that hypotonic fluid is associated with decreases in serum sodium levels in this population [90].

## 5. Conclusions

Current evidence supports the use of isotonic solution as maintenance IVF which could prevent iatrogenic hyponatraemia in hospitalised children and consequently avoid unfavourable incidents. However, because both isotonic and hypotonic fluids can cause hyponatraemia, fluid prescription should always be tailored to the specific needs of each child. According to our findings, the risk of adverse events is not substantially different between the two fluids; nevertheless, maintenance IVF should always be prescribed with care.

## Figures and Tables

**Table 1:** Characteristics of included RCTs of hypotonic vs. isotonic maintenance intravenous fluid therapy in hospitalised children.

No.	Study ID [References]	Country	Follow-Up Duration	Condition	Isotonic			Hypotonic		
					N	Age (Mean ± SD/Median (IQR)/Range)	Solution	N	Age (Mean ± SD/Median (IQR)/Range)	Solution
1	Almeida 2014 [41]	Portugal	24 h	Surgical and medical	130	49.9 ± 62.5 (months)	NaCl 0.9%, with 154 mEq Na and Cl/L in 5% dextrose	103	41.1 ± 64.4 (months)	NaCl 0.45%, with 75 mEq Na and Cl/L in 5% dextrose
2	Bagri 2019 [42]	India	48 h	Medical	74	36.0 (12.0–108.0) (months)	0.9% saline in 5% dextrose with 20 mEq/L of potassium chloride	74	60.0 (13.0–120.0) (months)	0.45% saline in 5% dextrose with 20 mEq/L of potassium chloride
3	Balasubramaniam 2011 [43]	India	24 h	Medical	42	4.9 ± 2.0 (days)	0.9% saline in 5% dextrose	42	5.5 ± 1.9 (days)	0.2% saline in 5% dextrose
4	Brazel 1996 [44]	Australia	NR	Surgical	5	12.3–18.1 (years)	Hartman’s solution	7	12.3–18.1 (years)	0.3% NS in 3% dextrose 0.18% NS in 4% dextrose
5	Choong 2011 [45]	Canada	48 h	Surgical	128	9.2 ± 5.5 (years)	0.9% saline in 5% dextrose	130	9.0 ± 5.7 (years)	0.45% saline in 5% dextrose
6	Coulthard 2012 [46]	Australia	18 h	Surgical	39	136.0 (52.0–167.0) (months)	Hartmann’s and 5% dextrose	40	138.0 (72.0–169.0) (months)	0.45% NaCl and 5% dextrose
7	Flores Robles 2015 [47]	Canada	8 h	Surgical and medical	52	58.8 ± 57.7 (months)	0.9% saline in 5% dextrose	49	63.5 ± 56.1 (months)	0.3% saline in 3.3% dextrose
								50	54.6 ± 55.9 (months)	0.45% saline in 5% dextrose
8	Friedman 2015 [48]	Canada	24 h	Medical	47	3.9 (2.0–6.9) (years)	0.9% saline in 5% dextrose	45	5.8 (1.4–11.2) (years)	0.45% saline in 5% dextrose
9	Jorro Baron 2013 [49]	Argentina	24 h	Surgical and medical	31	5.0 (3.0–9.0) (months)	154 mmol/L sodium + 20 mmol/L potassium in 5% dextrose	32	5.0 (3.0–10.0) (months)	77 mmol/L sodium + 20 mmol/L potassium in 5% dextrose
10	Kannan 2010 [40]	India	24 h	Medical	58	36.0 (12.0–84.0) (months)	0.9% saline in 5% dextrose at standard maintenance rate	56	48.0 (12.7–72.0) (months)	0.18% saline in 5% dextrose at the standard maintenance rate
								53	36.0 (10.0–66.0) (months)	0.18% saline in 5% dextrose at 2/3 of the standard maintenance rate
11	Kumar 2019 [50]	India	24 h	Medical	84	16.0 (7.0–30.0) (months)	0.9% saline in 5% dextrose	84	11.0 (5.0–28.5) (months)	0.45% saline in 5% dextrose
12	Lehtiranta 2020 [51]	Finland	7 days	Surgical and medical	308	4.0 ± 3.1 (years)	140 mmol/L of sodium and 5 mmol/L potassium in 5% dextrose	306	4.1 ± 3.1 (years)	80 mmol/L sodium and 20 mmol/L potassium in 5% dextrose
13	McNab 2014 [52]	Australia	72 h	Surgical	319	8.2 ± 5.4 (years)	140 mmol/L of sodium	322	8.9 ± 5.3 (years)	77 mmol/L of sodium
14	Mierzewska-Schmidt 2015 [53]	Poland	NR	Surgical	30	6.1 ± 2.1 (years)	Ringer’s acetate	33	6.2 ± 2.1 (years)	5% glucose in water solution
								27	6.5 ± 2.5 (years)	3.33% glucose in 0.3% NaCl
15	Montanana 2008 [54]	Spain	24 h	Surgical and medical	51	3.2 (1.3–10.0) (years)	140 mEq/L sodium + 15 mEq/L potassium in 5% dextrose	52	3.0 (0.9–7.0) (years)	20 and 100 mEq/L sodium in 5% dextrose
16	Omoifo 2018 [55]	Nigeria	NR	Surgical	20	5.9 ± 3.5 (years)	Normal saline	25	6.5 ± 3.7 (years)	4.3% dextrose in 0.18 saline
17	Pemde 2015 [56]	India	24 h	Medical	31	26.2 ± 19.6) (months)	0.9% saline in 5% dextrose	30	31.9 ± 20.7 (months)	0.45% saline in 5% dextrose
								31	28.2 ± 21.2 (months)	0.18% saline in 5% dextrose
18	Ramanathan 2015 [57]	India	24 h	Medical	59	2.0–60.0 (months)	0.9% saline in 5% dextrose and potassium chloride 20 meq/L	60	2.0–60.0 (months)	0.18% saline in 5% dextrose and potassium chloride 20 meq/L
19	Raksha 2017 [58]	India	24 h	Medical	120	1.0 month–18.0 years old	0.9% saline in 5% dextrose with 20 mEq/L of potassium chloride at standard maintenance rate	120	1.0 month–18.0 years old	0.18% saline in 5% dextrose/isolyte-p at 2/3 standard maintenance rate
20	Saba 2011 [59]	Canada	8 h	Surgical and medical	16	8.2 (2.8–14.3) (years)	0.9% saline in 5% dextrose	21	8.9 (1.7–16.5) (years)	0.45% saline in 5% dextrose
21	Shamim 2014 [60]	India	48 h	Medical	30	53.1 ± 39.5 (months)	0.9% NaCl in 5% dextrose at the rate of 60% of standard maintenance volume	30	54.4 ± 31.7 (months)	0.18% NaCl in 5% dextrose at the rate of standard maintenance volume
22	Torres 2019 [61]	Argentina	24 h	Surgical and medical	145	18.0 (2.0–110.0) (months)	0.9% saline in 5% dextrose	154	21.0 (3.0–109.0) (months)	0.45% saline in 5% dextrose

NR: not reported.

## Data Availability

The data presented in this study are available within the article and Supplementary Materials.

## Supplementary Materials

The following are available online at https://www.mdpi.com/article/10.3390/children8090785/s1, Table S1: Database search strategies, Table S2: Sensitivity analyses, Table S3: Quality assessment of the included studies, Figure S1: Risk of developing hyponatraemia followed by isotonic vs. hypotonic fluids in hospitalised children, Figure S2: Prevalence of hyponatraemia (A,B) and hypernatraemia (C,D) following isotonic and hypotonic fluids in hospitalised children at ≤24 and >24 h, Figure S3: Risk of developing hyponatraemia followed by isotonic (0.9%) vs. hypotonic fluids (0.18% or 0.45%) in hospitalised children, Figure S4: Mean differences of serum sodium levels (A,B), urine sodium (C,D), and serum osmolarity/osmolality (E,F) following isotonic and hypotonic fluids in hospitalised children at ≤24 and >24 h, Figure S5: Prevalence of adverse events following isotonic and hypotonic fluids in hospitalised children, Figure S6: Risk of adverse events following isotonic and hypotonic fluids in hospitalised children, Figure S7: Funnel plots representing significant publication bias in estimating the prevalence of hyponatraemia followed by (A) isotonic and (B) hypotonic fluids and hypernatraemia followed by (C) isotonic and (D) hypotonic fluids.
