# Supplementary material for: Efficacy and Safety of Isotonic and Hypotonic Intravenous Maintenance Fluids in Hospitalised Children: A Systematic Review and Meta-Analysis of Randomised Controlled Trials
Source: Children (Basel). 2021 Sep 8;8(9):785. doi: 10.3390/children8090785 (PMC8471545; doi:10.3390/children8090785)
Supplement: Supplementary file 1 [file children-08-00785-s001.zip › Figure S1_Hyponatremia_Differnt time points.pdf]

(A) Hyponatraemia, 6hrs

Study or Subgroup	Isotonic Events	Isotonic Total	Hypotonic Events	Hypotonic Total	Weight	Risk Ratio M-H, Random, 95% CI
Montanana 2008	15	51	20	52	53.8%	0.76 [0.44, 1.32]
Ramanathan 2015	6	59	11	60	30.0%	0.55 [0.22, 1.40]
Pemde 2015	2	31	18	61	16.2%	0.22 [0.05, 0.88]
**Total (95% CI)**		**141**		**173**	**100.0%**	**0.57 [0.31, 1.05]**
Total events	23		49			

Heterogeneity: Tau² = 0.11; Chi² = 3.02, df = 2 (P = 0.22); I² = 34%
Test for overall effect: Z = 1.80 (P = 0.07)

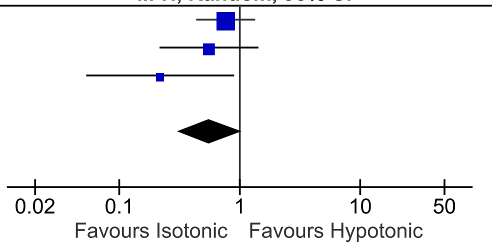

(B) Hyponatraemia, 8hrs

Study or Subgroup	Isotonic Events	Isotonic Total	Hypotonic Events	Hypotonic Total	Weight	Risk Ratio M-H, Random, 95% CI
Saba 2011	1	16	1	21	14.4%	1.31 [0.09, 19.42]
Balasubramaniam 2011	4	42	20	42	61.1%	0.20 [0.07, 0.54]
Flores Robles 2015	1	34	21	64	24.5%	0.09 [0.01, 0.64]
**Total (95% CI)**		**92**		**127**	**100.0%**	**0.22 [0.07, 0.64]**
Total events	6		42			

Heterogeneity: Tau² = 0.25; Chi² = 2.60, df = 2 (P = 0.27); I² = 23%
Test for overall effect: Z = 2.77 (P = 0.006)

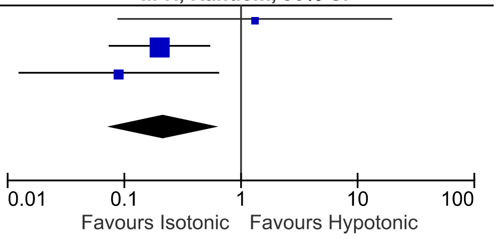

(C) Hyponatraemia, 12hrs

Study or Subgroup	Isotonic Events	Isotonic Total	Hypotonic Events	Hypotonic Total	Weight	Risk Ratio M-H, Random, 95% CI
Kumar 2019	4	84	5	84	16.8%	0.80 [0.22, 2.88]
Shamim 2014	3	30	4	30	14.5%	0.75 [0.18, 3.07]
Torres 2019	12	145	29	154	40.1%	0.44 [0.23, 0.83]
Pemde 2015	3	31	26	61	20.7%	0.23 [0.07, 0.69]
Ramanathan 2015	1	59	12	60	7.9%	0.08 [0.01, 0.63]
**Total (95% CI)**		**349**		**389**	**100.0%**	**0.40 [0.22, 0.73]**
Total events	23		76			

Heterogeneity: Tau² = 0.13; Chi² = 5.50, df = 4 (P = 0.24); I² = 27%
Test for overall effect: Z = 2.98 (P = 0.003)

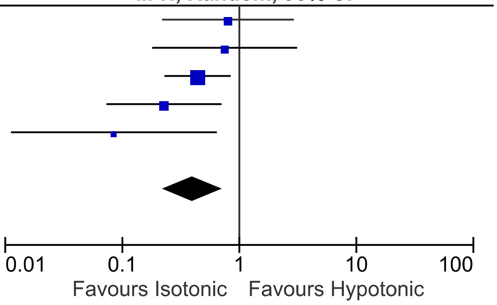

(D) Hyponatraemia, 18hrs

Study or Subgroup	Isotonic Events	Isotonic Total	Hypotonic Events	Hypotonic Total	Weight	Risk Ratio M-H, Random, 95% CI
Pemde 2015	3	31	24	61	86.5%	0.25 [0.08, 0.75]
Coulthard 2012	0	39	7	40	13.5%	0.07 [0.00, 1.16]
**Total (95% CI)**		**70**		**101**	**100.0%**	**0.21 [0.07, 0.59]**
Total events	3		31			

Heterogeneity: Tau² = 0.00; Chi² = 0.71, df = 1 (P = 0.40); I² = 0%
Test for overall effect: Z = 2.97 (P = 0.003)

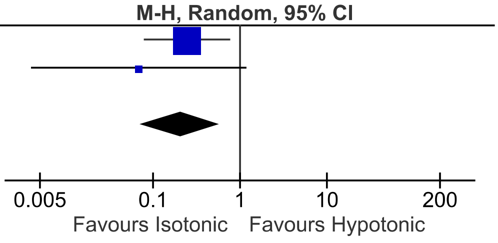

(E) Hyponatraemia, 24hrs

Study or Subgroup	Isotonic Events	Isotonic Total	Hypotonic Events	Hypotonic Total	Weight	Risk Ratio M-H, Random, 95% CI
Jorro Baron 2013	4	31	5	32	4.3%	0.83 [0.24, 2.79]
Kannan 2010	5	58	18	109	7.3%	0.52 [0.20, 1.33]
Bagri 2019	3	75	6	75	3.5%	0.50 [0.13, 1.93]
Raksha 2017	9	120	20	120	11.6%	0.45 [0.21, 0.95]
Shamim 2014	7	30	16	30	12.1%	0.44 [0.21, 0.91]
Kumar 2019	5	84	12	84	6.5%	0.42 [0.15, 1.13]
Almeida 2014	7	130	14	103	8.5%	0.40 [0.17, 0.95]
Ramanathan 2015	2	59	6	60	2.7%	0.34 [0.07, 1.61]
Torres 2019	18	145	71	154	29.8%	0.27 [0.17, 0.43]
Montanana 2008	3	23	13	23	5.2%	0.23 [0.08, 0.70]
Balasubramaniam 2011	3	42	18	42	4.9%	0.17 [0.05, 0.52]
Pemde 2015	2	31	29	61	3.5%	0.14 [0.03, 0.53]
**Total (95% CI)**		**828**		**893**	**100.0%**	**0.35 [0.27, 0.45]**
Total events	68		228			

Heterogeneity: Tau² = 0.00; Chi² = 9.21, df = 11 (P = 0.60); I² = 0%
Test for overall effect: Z = 8.20 (P < 0.00001)

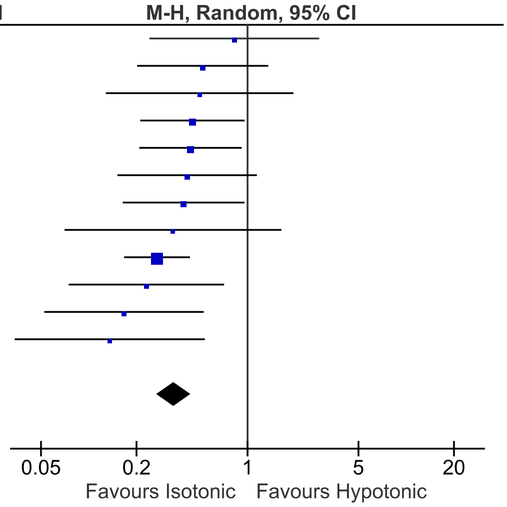

(F) Hyponatraemia, 36hrs

Study or Subgroup	Isotonic Events	Isotonic Total	Hypotonic Events	Hypotonic Total	Weight	Risk Ratio M-H, Random, 95% CI
Shamim 2014	3	30	14	30	100.0%	0.21 [0.07, 0.67]
**Total (95% CI)**		**30**		**30**	**100.0%**	**0.21 [0.07, 0.67]**
Total events	3		14			

Heterogeneity: Not applicable
Test for overall effect: Z = 2.65 (P = 0.008)

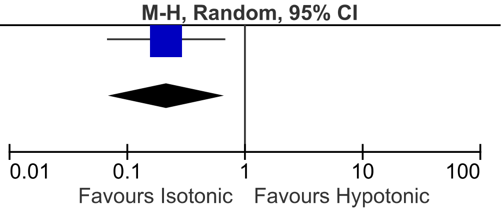

(G) Hyponatraemia, 48hrs

Study or Subgroup	Isotonic Events	Isotonic Total	Hypotonic Events	Hypotonic Total	Weight	Risk Ratio M-H, Random, 95% CI
Bagri 2019	5	75	6	75	9.9%	0.83 [0.27, 2.61]
Choong 2011	26	128	47	130	75.8%	0.56 [0.37, 0.85]
Shamim 2014	4	30	13	30	12.9%	0.31 [0.11, 0.84]
Friedman 2015	0	47	2	45	1.4%	0.19 [0.01, 3.89]
**Total (95% CI)**		**280**		**280**	**100.0%**	**0.53 [0.37, 0.76]**
Total events	35		68			

Heterogeneity: Tau² = 0.00; Chi² = 2.26, df = 3 (P = 0.52); I² = 0%
Test for overall effect: Z = 3.44 (P = 0.0006)

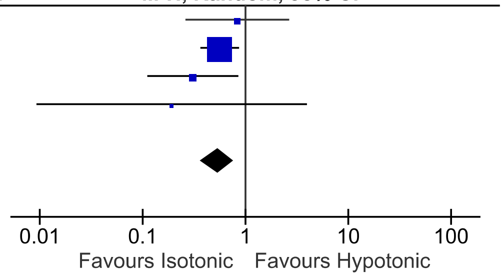

(H) Hyponatraemia, 72hrs

Study or Subgroup	Isotonic Events	Isotonic Total	Hypotonic Events	Hypotonic Total	Weight	Risk Ratio M-H, Random, 95% CI
McNab 2014	12	319	35	322	82.8%	0.35 [0.18, 0.65]
Brazel 1996	1	5	7	7	17.2%	0.27 [0.07, 1.08]
**Total (95% CI)**		**324**		**329**	**100.0%**	**0.33 [0.19, 0.59]**
Total events	13		42			

Heterogeneity: Tau² = 0.00; Chi² = 0.11, df = 1 (P = 0.74); I² = 0%
Test for overall effect: Z = 3.74 (P = 0.0002)

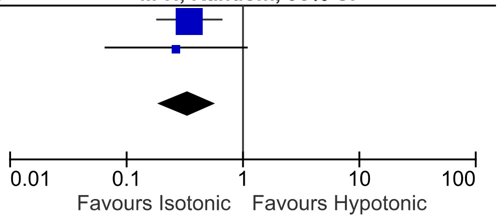

(I) Hyponatraemia, 7 days

Study or Subgroup	Isotonic Events	Isotonic Total	Hypotonic Events	Hypotonic Total	Weight	Risk Ratio M-H, Random, 95% CI
Lehtiranta 2020	7	308	11	306	100.0%	0.63 [0.25, 1.61]
**Total (95% CI)**		**308**		**306**	**100.0%**	**0.63 [0.25, 1.61]**
Total events	7		11			

Heterogeneity: Not applicable
Test for overall effect: Z = 0.96 (P = 0.34)

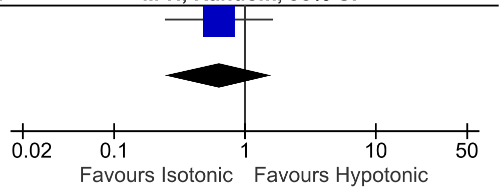

Figure S1. Risk of developing hyponatraemia followed by isotonic vs hypotonic fluids in hospitalised children.
