# Supplementary material for: Efficacy and Safety of Isotonic and Hypotonic Intravenous Maintenance Fluids in Hospitalised Children: A Systematic Review and Meta-Analysis of Randomised Controlled Trials
Source: Children (Basel). 2021 Sep 8;8(9):785. doi: 10.3390/children8090785 (PMC8471545; doi:10.3390/children8090785)
Supplement: Supplementary file 1 [file children-08-00785-s001.zip › Figure S2_Prev_hypo+hyper_R.pdf]

**A**

| Study ID | Cases | Total | Prevalence | 95% C.I. |
|---|---|---|---|---|
| **Hyponatraemia (Isotonic, ≤24 hours)** | | | | |
| Almeida 2014 | 7 | 130 | 5.4 | [1.5; 9.3] |
| Bagri 2019 | 3 | 75 | 4.0 | [0.0; 8.4] |
| Balasubramaniam 2011 | 3 | 42 | 7.1 | [0.0; 14.9] |
| Coulthard 2012 | 0 | 39 | 0.0 | [0.0; 3.4] |
| Flores Robles 2015 | 1 | 34 | 2.9 | [0.0; 8.6] |
| Jorro Baron 2013 | 4 | 31 | 12.9 | [1.1; 24.7] |
| Kannan 2010 | 5 | 58 | 8.6 | [1.4; 15.8] |
| Kumar 2019 | 5 | 84 | 6.0 | [0.9; 11.0] |
| Mierzewska-Schmidt 2015 | 1 | 30 | 3.3 | [0.0; 9.8] |
| Montanana 2008 | 3 | 23 | 13.0 | [0.0; 26.8] |
| Omoifo 2018 | 1 | 40 | 2.5 | [0.0; 7.3] |
| Pemde 2015 | 2 | 31 | 6.5 | [0.0; 15.1] |
| Raksha 2017 | 9 | 120 | 7.5 | [2.8; 12.2] |
| Ramanathan 2015 | 2 | 59 | 3.4 | [0.0; 8.0] |
| Saba 2011 | 1 | 16 | 6.2 | [0.0; 18.1] |
| Shamim 2014 | 7 | 30 | 23.3 | [8.2; 38.5] |
| Torres 2019 | 18 | 145 | 12.4 | [7.0; 17.8] |
| **Random effects model** | | **987** | **5.7** | **[3.7; 7.6]** |

Heterogeneity: $I^2 = 45\%$, $\tau^2 = 0.0007$, $\chi^2_{16} = 29.21$ ($p = 0.02$)

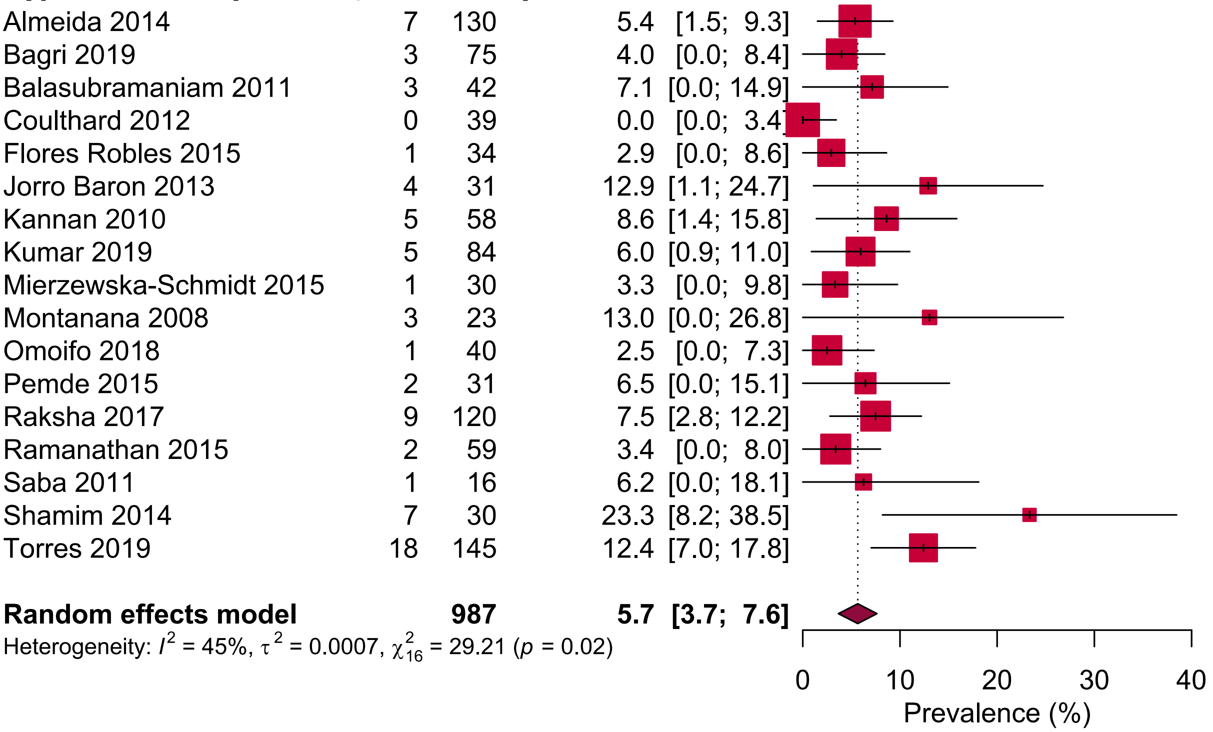


**B**

| Study ID | Cases | Total | Prevalence | 95% C.I. |
|---|---|---|---|---|
| **Hyponatraemia (Hypotonic, ≤24 hours)** | | | | |
| Almeida 2014 | 14 | 103 | 13.6 | [ 7.0; 20.2] |
| Bagri 2019 | 6 | 75 | 8.0 | [ 1.9; 14.1] |
| Balasubramaniam 2011 | 18 | 42 | 42.9 | [27.9; 57.8] |
| Coulthard 2012 | 7 | 40 | 17.5 | [ 5.7; 29.3] |
| Flores Robles 2015 | 21 | 64 | 32.8 | [21.3; 44.3] |
| Jorro Baron 2013 | 5 | 32 | 15.6 | [ 3.0; 28.2] |
| Kannan 2010 | 18 | 109 | 16.5 | [ 9.5; 23.5] |
| Kumar 2019 | 12 | 84 | 14.3 | [ 6.8; 21.8] |
| Mierzewska-Schmidt 2015 | 12 | 60 | 20.0 | [ 9.9; 30.1] |
| Montanana 2008 | 13 | 23 | 56.5 | [36.3; 76.8] |
| Omoifo 2018 | 1 | 25 | 4.0 | [ 0.0; 11.7] |
| Pemde 2015 | 29 | 61 | 47.5 | [35.0; 60.1] |
| Raksha 2017 | 20 | 120 | 16.7 | [10.0; 23.3] |
| Ramanathan 2015 | 6 | 60 | 10.0 | [ 2.4; 17.6] |
| Saba 2011 | 1 | 21 | 4.8 | [ 0.0; 13.9] |
| Shamim 2014 | 16 | 30 | 53.3 | [35.5; 71.2] |
| Torres 2019 | 71 | 154 | 46.1 | [38.2; 54.0] |
| **Random effects model** | | **1103** | **23.3** | **[16.4; 30.1]** |

Heterogeneity: $I^2 = 90\%$, $\tau^2 = 0.0178$, $\chi^2_{16} = 154.77$ ($p < 0.01$)

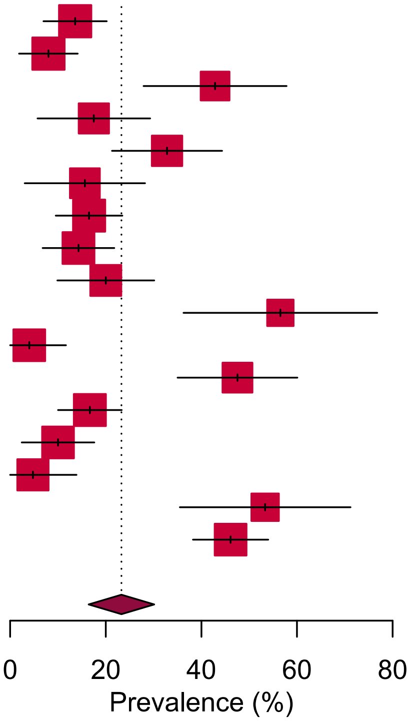

**C**

| Study ID | Cases | Total | Prevalence | 95% C.I. |
|---|---|---|---|---|
| **Hyponatraemia (Isotonic, >24 hours)** | | | | |
| Bagri 2019 | 5 | 75 | 6.7 | [ 1.0; 12.3] |
| Brazel 1996 | 1 | 5 | 20.0 | [ 0.0; 55.1] |
| Choong 2011 | 26 | 128 | 20.3 | [13.3; 27.3] |
| Friedman 2015 | 0 | 47 | 0.0 | [ 0.0; 2.9] |
| Lehtiranta 2020 | 7 | 308 | 2.3 | [ 0.6; 3.9] |
| McNab 2014 | 12 | 319 | 3.8 | [ 1.7; 5.8] |
| Shamim 2014 | 4 | 30 | 13.3 | [ 1.2; 25.5] |
| **Random effects model** | | **912** | **6.0** | **[ 2.3; 9.6]** |

Heterogeneity: $I^2 = 83\%$, $\tau^2 = 0.0014$, $\chi^2_6 = 34.38$ ($p < 0.01$)

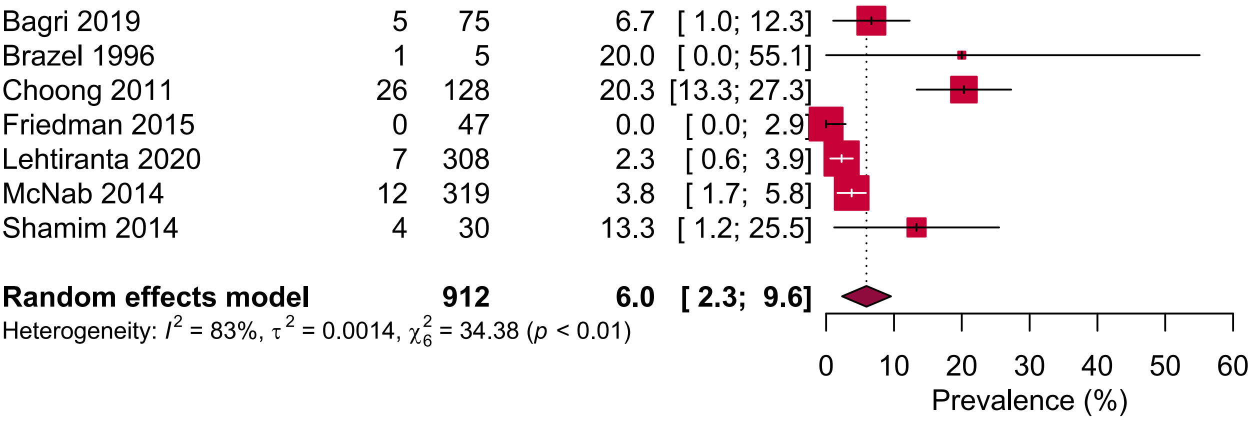


**D**

| Study ID | Cases | Total | Prevalence | 95% C.I. |
|---|---|---|---|---|
| **Hyponatraemia (Hypotonic, >24 hours)** | | | | |
| Bagri 2019 | 6 | 75 | 8.0 | [ 1.9; 14.1] |
| Brazel 1996 | 7 | 7 | 100.0 | [83.2; 100.0] |
| Choong 2011 | 47 | 130 | 36.2 | [27.9; 44.4] |
| Friedman 2015 | 2 | 45 | 4.4 | [ 0.0; 10.5] |
| Lehtiranta 2020 | 11 | 306 | 3.6 | [ 1.5; 5.7] |
| McNab 2014 | 35 | 322 | 10.9 | [ 7.5; 14.3] |
| Shamim 2014 | 13 | 30 | 43.3 | [25.6; 61.1] |
| **Random effects model** | | **915** | **26.3** | **[14.9; 37.7]** |

Heterogeneity: $I^2 = 97\%$, $\tau^2 = 0.0212$, $\chi^2_6 = 196.67$ ($p < 0.01$)

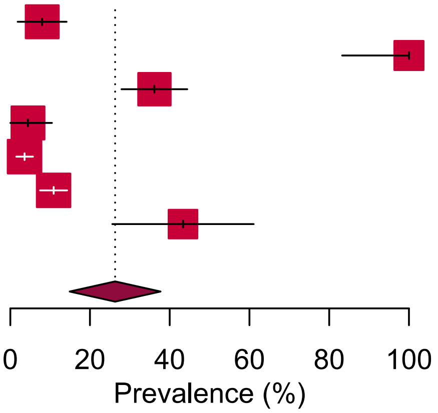

**E**

| Study ID | Cases | Total | Prevalence | 95% C.I. |
|---|---|---|---|---|
| **Hypernatraemia (Isotonic, ≤24 hours)** | | | | |
| Almeida 2014 | 10 | 130 | 7.7 | [ 3.1; 12.3] |
| Bagri 2019 | 3 | 75 | 4.0 | [ 0.0; 8.4] |
| Balasubramaniam 2011 | 14 | 42 | 33.3 | [19.1; 47.6] |
| Flores Robles 2015 | 2 | 52 | 3.8 | [ 0.0; 9.1] |
| Jorro Baron 2013 | 1 | 31 | 3.2 | [ 0.0; 9.4] |
| Kannan 2010 | 2 | 58 | 3.4 | [ 0.0; 8.1] |
| Kumar 2019 | 3 | 84 | 3.6 | [ 0.0; 7.5] |
| Mierzewska-Schmidt 2015 | 1 | 30 | 3.3 | [ 0.0; 9.8] |
| Montanana 2008 | 1 | 23 | 4.3 | [ 0.0; 12.7] |
| Pemde 2015 | 1 | 31 | 3.2 | [ 0.0; 9.4] |
| Raksha 2017 | 3 | 120 | 2.5 | [ 0.0; 5.3] |
| Ramanathan 2015 | 0 | 59 | 0.0 | [ 0.0; 2.3] |
| Saba 2011 | 1 | 16 | 6.2 | [ 0.0; 18.1] |
| **Random effects model** | | **751** | **4.0** | **[ 1.9; 6.2]** |

Heterogeneity: $I^2 = 58\%$, $\tau^2 = 0.0008$, $\chi^2_{12} = 28.88$ ($p < 0.01$)

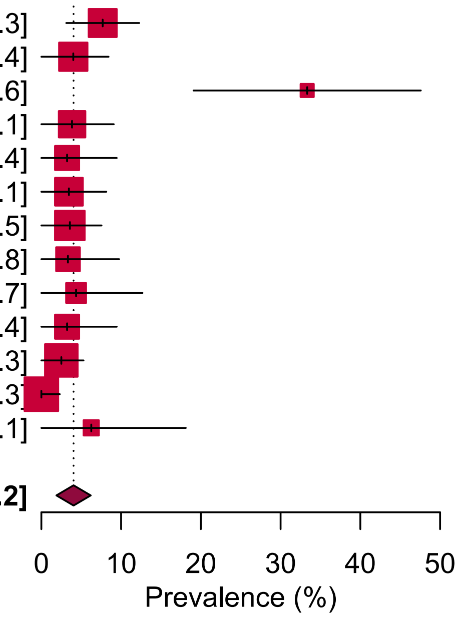


**F**

| Study ID | Cases | Total | Prevalence | 95% C.I. |
|---|---|---|---|---|
| **Hypernatraemia (Hypotonic, ≤24 hours)** | | | | |
| Almeida 2014 | 2 | 103 | 1.9 | [0.0; 4.6] |
| Bagri 2019 | 1 | 75 | 1.3 | [0.0; 3.9] |
| Balasubramaniam 2011 | 4 | 42 | 9.5 | [0.6; 18.4] |
| Flores Robles 2015 | 0 | 99 | 0.0 | [0.0; 1.4] |
| Jorro Baron 2013 | 2 | 32 | 6.2 | [0.0; 14.6] |
| Kannan 2010 | 6 | 109 | 5.5 | [1.2; 9.8] |
| Kumar 2019 | 4 | 84 | 4.8 | [0.2; 9.3] |
| Mierzewska-Schmidt 2015 | 0 | 60 | 0.0 | [0.0; 2.3] |
| Montanana 2008 | 1 | 23 | 4.3 | [0.0; 12.7] |
| Pemde 2015 | 0 | 61 | 0.0 | [0.0; 2.2] |
| Raksha 2017 | 0 | 120 | 0.0 | [0.0; 1.1] |
| Ramanathan 2015 | 0 | 60 | 0.0 | [0.0; 2.3] |
| Saba 2011 | 0 | 21 | 0.0 | [0.0; 6.2] |
| **Random effects model** | | **889** | **0.9** | **[0.0; 1.9]** |

Heterogeneity: $I^2 = 35\%$, $\tau^2 < 0.0001$, $\chi^2_{12} = 18.54$ ($p = 0.10$)

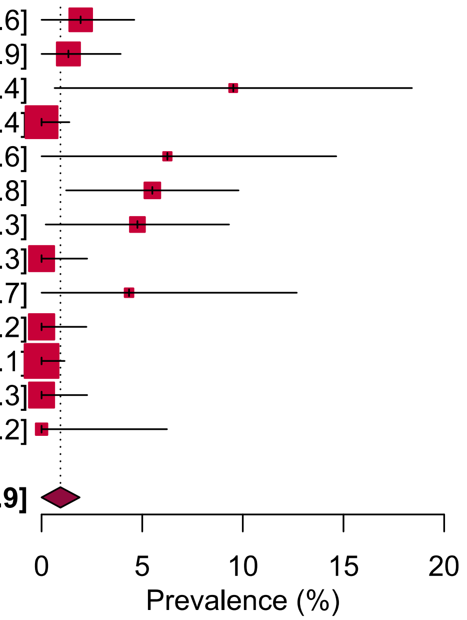

**G**

| Study ID | Cases | Total | Prevalence | 95% C.I. |
|---|---|---|---|---|
| **Hypernatraemia (Isotonic, >24 hours)** | | | | |
| Bagri 2019 | 3 | 75 | 4.0 | [0.0; 8.4] |
| Choong 2011 | 3 | 128 | 2.3 | [0.0; 5.0] |
| Friedman 2015 | 1 | 47 | 2.1 | [0.0; 6.3] |
| Lehtiranta 2020 | 4 | 308 | 1.3 | [0.0; 2.6] |
| McNab 2014 | 14 | 319 | 4.4 | [2.1; 6.6] |
| Shamim 2014 | 3 | 30 | 10.0 | [0.0; 20.7] |
| **Random effects model** | | **907** | **2.7** | **[1.2; 4.2]** |

Heterogeneity: $I^2 = 39\%$, $\tau^2 = 0.0001$, $\chi^2_5 = 8.26$ ($p = 0.14$)

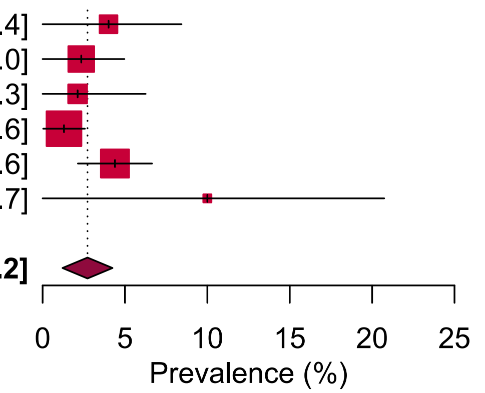


**H**

| Study ID | Cases | Total | Prevalence | 95% C.I. |
|---|---|---|---|---|
| **Hypernatraemia (Hypotonic, >24 hours)** | | | | |
| Bagri 2019 | 1 | 75 | 1.3 | [0.0; 3.9] |
| Choong 2011 | 4 | 130 | 3.1 | [0.1; 6.0] |
| Friedman 2015 | 1 | 45 | 2.2 | [0.0; 6.5] |
| Lehtiranta 2020 | 0 | 306 | 0.0 | [0.0; 0.5] |
| McNab 2014 | 18 | 322 | 5.6 | [3.1; 8.1] |
| Shamim 2014 | 0 | 30 | 0.0 | [0.0; 4.4] |
| **Random effects model** | | **908** | **2.0** | **[0.0; 4.1]** |

Heterogeneity: $I^2 = 79\%$, $\tau^2 = 0.0005$, $\chi^2_5 = 23.52$ ($p < 0.01$)

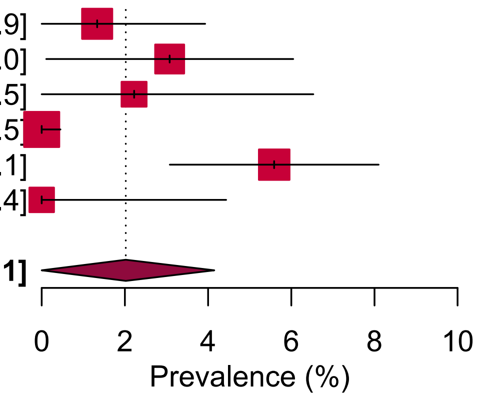


**Figure S2.** Prevalence of hyponatraemia (A-B) and hypernatraemia (C-D) following isotonic and hypotonic fluids in hospitalised children at ≤ 24 hours and > 24 hours
