# Supplementary material for: Efficacy and Safety of Isotonic and Hypotonic Intravenous Maintenance Fluids in Hospitalised Children: A Systematic Review and Meta-Analysis of Randomised Controlled Trials
Source: Children (Basel). 2021 Sep 8;8(9):785. doi: 10.3390/children8090785 (PMC8471545; doi:10.3390/children8090785)
Supplement: Supplementary file 1 [file children-08-00785-s001.zip › Figure S3_Hypo_0.9% vs 0.18% or 0.45%.pdf]

(A) Hyponatraemia, 24 hrs, 0.9% vs 0.18%

Study or Subgroup	Isotonic Events	Isotonic Total	Hypotonic Events	Hypotonic Total	Weight	Risk Ratio M-H, Random, 95% CI
Kannan 2010	5	58	18	109	19.7%	0.52 [0.20, 1.33]
Pemde 2015	2	31	17	31	9.2%	0.12 [0.03, 0.47]
Raksha 2017	9	120	20	120	31.3%	0.45 [0.21, 0.95]
Ramanathan 2015	2	59	6	60	7.1%	0.34 [0.07, 1.61]
Shamim 2014	7	30	16	30	32.6%	0.44 [0.21, 0.91]
**Total (95% CI)**		**298**		**350**	**100.0%**	**0.40 [0.26, 0.60]**
Total events	25		77			

Heterogeneity: Tau² = 0.00; Chi² = 3.64, df = 4 (P = 0.46); I² = 0%

Test for overall effect: Z = 4.33 (P < 0.0001)

Risk Ratio M-H, Random, 95% CI

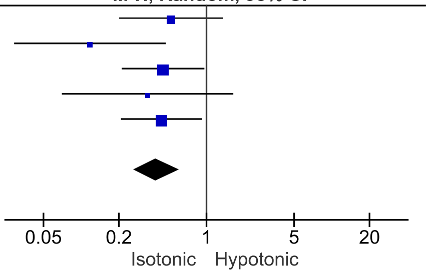

(B) Hyponatraemia, 24 hrs, 0.9% vs 0.45%

Study or Subgroup	Isotonic Events	Isotonic Total	Hypotonic Events	Hypotonic Total	Weight	Risk Ratio M-H, Random, 95% CI
Almeida 2014	7	130	14	103	16.5%	0.40 [0.17, 0.95]
Bagri 2019	3	75	6	75	6.9%	0.50 [0.13, 1.93]
Kumar 2019	5	84	12	84	12.5%	0.42 [0.15, 1.13]
Pemde 2015	2	31	12	30	6.3%	0.16 [0.04, 0.66]
Torres 2019	18	145	71	154	57.8%	0.27 [0.17, 0.43]
**Total (95% CI)**		**465**		**446**	**100.0%**	**0.31 [0.22, 0.44]**
Total events	35		115			

Heterogeneity: Tau² = 0.00; Chi² = 2.30, df = 4 (P = 0.68); I² = 0%

Test for overall effect: Z = 6.56 (P < 0.00001)

Risk Ratio M-H, Random, 95% CI

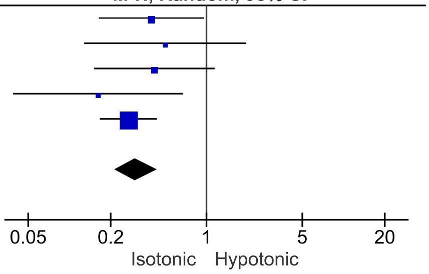

Figure S3. Risk of developing hyponatraemia followed by isotonic (0.9%) vs hypotonic fluids (0.18% or 0.45%) in hospitalised children.
