# Supplementary material for: Efficacy and Safety of Isotonic and Hypotonic Intravenous Maintenance Fluids in Hospitalised Children: A Systematic Review and Meta-Analysis of Randomised Controlled Trials
Source: Children (Basel). 2021 Sep 8;8(9):785. doi: 10.3390/children8090785 (PMC8471545; doi:10.3390/children8090785)
Supplement: Supplementary file 1 [file children-08-00785-s001.zip › Figure S4_Serum Sodium, serum osmolarity, urine sodium.pdf]

(A) Serum Sodium, ≤24hrs

Study or Subgroup	Isotonic Mean [mEq/L]	Isotonic SD [mEq/L]	Isotonic Total	Hypotonic Mean [mEq/L]	Hypotonic SD [mEq/L]	Hypotonic Total	Weight	Mean Difference IV, Random, 95% CI
Almeida 2014	140	4.9	130	137.8	3	103	7.8%	2.20 [1.18, 3.22]
Bagri 2019	138.3	6	75	135.1	4.4	75	6.8%	3.20 [1.52, 4.88]
Coulthard 2012	138.1	1.9	39	136.7	2.7	40	7.8%	1.40 [0.37, 2.43]
Flores Robles 2015	137.9	2.8	52	134.8	2.1	99	8.0%	3.10 [2.23, 3.97]
Friedman 2015	140.5	2.7	47	139.6	2.7	45	7.7%	0.90 [-0.20, 2.00]
Jorro Baron 2013	140	4.1	31	137.8	4.3	32	6.1%	2.20 [0.13, 4.27]
Kumar 2019	139.3	4.4	84	137.6	4	84	7.4%	1.70 [0.43, 2.97]
McNab 2014	139.9	3.4	145	139.7	3.4	163	8.1%	0.20 [-0.56, 0.96]
Mierzewska-Schmidt 2015	141.4	1.8	30	136.4	3.2	60	7.8%	5.00 [3.97, 6.03]
Montanana 2008	138.9	3.6	23	136.2	5.2	23	5.3%	2.70 [0.12, 5.28]
Omoifo 2018	134.3	5.7	40	134.2	6.3	25	4.6%	0.10 [-2.94, 3.14]
Raksha 2017	138	3	120	136.7	3.2	120	8.1%	1.30 [0.52, 2.08]
Ramanathan 2015	143.6	4.8	59	138	4.6	60	6.8%	5.60 [3.91, 7.29]
Shamim 2014	134.6	2.1	30	129.7	1.4	30	7.9%	4.90 [4.00, 5.80]
**Total (95% CI)**			**905**			**959**	**100.0%**	**2.50 [1.53, 3.46]**

Heterogeneity: Tau² = 2.84; Chi² = 124.64, df = 13 (P < 0.00001); I² = 90%
Test for overall effect: Z = 5.08 (P < 0.00001)

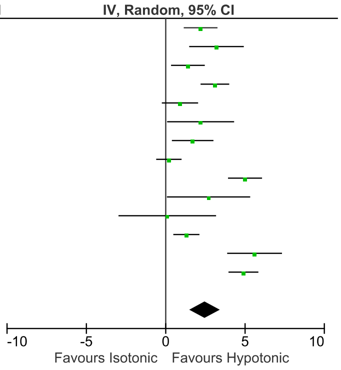

(B) Serum Sodium, >24hrs

Study or Subgroup	Isotonic Mean [mEq/L]	Isotonic SD [mEq/L]	Isotonic Total	Hypotonic Mean [mEq/L]	Hypotonic SD [mEq/L]	Hypotonic Total	Weight	Mean Difference IV, Random, 95% CI
Bagri 2019	137.4	6	75	138.1	6	75	24.6%	-0.70 [-2.62, 1.22]
Friedman 2015	139.9	2.7	30	139.6	2.6	34	25.4%	0.30 [-1.00, 1.60]
McNab 2014	140.3	3.5	25	139.3	3.8	29	24.6%	1.00 [-0.95, 2.95]
Shamim 2014	138.9	2.9	30	131.4	2.2	30	25.4%	7.50 [6.20, 8.80]
**Total (95% CI)**			**160**			**168**	**100.0%**	**2.05 [-2.00, 6.11]**

Heterogeneity: Tau² = 16.41; Chi² = 80.17, df = 3 (P < 0.00001); I² = 96%
Test for overall effect: Z = 0.99 (P = 0.32)

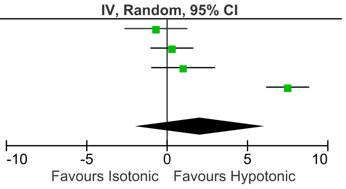

(C) Urine Sodium, ≤24hrs

Study or Subgroup	Isotonic Mean [mmol/L]	Isotonic SD [mmol/L]	Isotonic Total	Hypotonic Mean [mmol/L]	Hypotonic SD [mmol/L]	Hypotonic Total	Weight	Mean Difference IV, Random, 95% CI
Almeida 2014	106.6	56.8	130	81.8	5	103	27.8%	24.80 [14.99, 34.61]
Choong 2011	156.8	64.7	128	89	58.3	130	26.1%	67.80 [52.76, 82.84]
Coulthard 2012	136.3	76.8	39	113.8	73.7	40	18.4%	22.50 [-10.71, 55.71]
McNab 2014	138.4	80.3	319	79.5	45.8	322	27.7%	58.90 [48.77, 69.03]
**Total (95% CI)**			**616**			**595**	**100.0%**	**45.05 [21.70, 68.39]**

Heterogeneity: Tau² = 485.06; Chi² = 34.10, df = 3 (P < 0.00001); I² = 91%
Test for overall effect: Z = 3.78 (P = 0.0002)

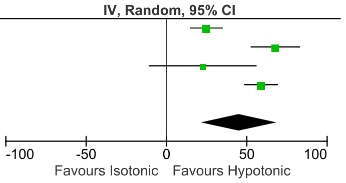

(D) Urine Sodium, >24hrs

Study or Subgroup	Isotonic Mean [mmol/L]	Isotonic SD [mmol/L]	Isotonic Total	Hypotonic Mean [mmol/L]	Hypotonic SD [mmol/L]	Hypotonic Total	Weight	Mean Difference IV, Random, 95% CI
Balasubramaniam 2011	66.6	26.8	42	28.2	13.6	42	52.3%	38.40 [29.31, 47.49]
Choong 2011	157.1	63.1	128	91.5	57.2	130	47.7%	65.60 [50.90, 80.30]
**Total (95% CI)**			**170**			**172**	**100.0%**	**51.36 [24.73, 77.99]**

Heterogeneity: Tau² = 331.03; Chi² = 9.51, df = 1 (P = 0.002); I² = 89%
Test for overall effect: Z = 3.78 (P = 0.0002)

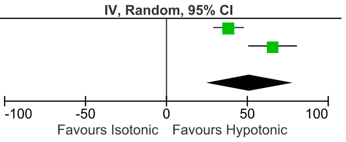

(E) Serum Osmolarity/Osmolality, ≤24hrs

Study or Subgroup	Isotonic Mean	Isotonic SD	Isotonic Total	Hypotonic Mean	Hypotonic SD	Hypotonic Total	Weight	Mean Difference IV, Random, 95% CI
Bagri 2019	290.9	14.1	75	268	15.1	75	23.3%	22.90 [18.22, 27.58]
Balasubramaniam 2011	286.6	5	42	282	5	42	25.6%	4.60 [2.46, 6.74]
Coulthard 2012	284.6	4.8	39	282.9	6.5	40	25.4%	1.70 [-0.82, 4.22]
Shamim 2014	290.9	4.8	30	279.8	3.2	30	25.7%	11.10 [9.04, 13.16]
**Total (95% CI)**			**186**			**187**	**100.0%**	**9.80 [3.12, 16.48]**

Heterogeneity: Tau² = 44.11; Chi² = 80.85, df = 3 (P < 0.00001); I² = 96%
Test for overall effect: Z = 2.88 (P = 0.004)

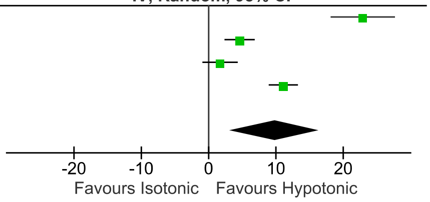

(F) Serum Osmolarity/Osmolality, >24hrs

Study or Subgroup	Isotonic Mean	Isotonic SD	Isotonic Total	Hypotonic Mean	Hypotonic SD	Hypotonic Total	Weight	Mean Difference IV, Random, 95% CI
Bagri 2019	271.3	13.3	75	266.4	8.9	75	49.6%	4.90 [1.28, 8.52]
Shamim 2014	300.7	5.9	30	282.2	4.4	30	50.4%	18.50 [15.87, 21.13]
**Total (95% CI)**			**105**			**105**	**100.0%**	**11.76 [-1.57, 25.09]**

Heterogeneity: Tau² = 89.87; Chi² = 35.43, df = 1 (P < 0.00001); I² = 97%
Test for overall effect: Z = 1.73 (P = 0.08)

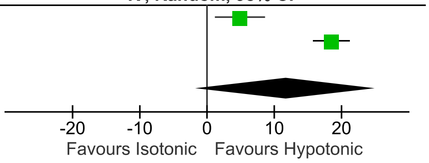

Figure S4. Mean differences of serum sodium levels (A-B), urine sodium (C-D) and serum osmolarity/osmolality (E-F) following isotonic and hypotonic fluids in hospitalised children at ≤ 24 hours and > 24 hours.
