# Supplementary material for: Efficacy and Safety of Isotonic and Hypotonic Intravenous Maintenance Fluids in Hospitalised Children: A Systematic Review and Meta-Analysis of Randomised Controlled Trials
Source: Children (Basel). 2021 Sep 8;8(9):785. doi: 10.3390/children8090785 (PMC8471545; doi:10.3390/children8090785)
Supplement: Supplementary file 1 [file children-08-00785-s001.zip › Figure S5_Adverse events_R.pdf]

A

Study ID	Cases	Total	Prevalence	95% C.I.
**Seizure (Isotonic)**				
Bagri 2019	0	75	0.0	[0; 1.8]
Flores Robles 2015	0	52	0.0	[0; 2.6]
Kannan 2010	0	58	0.0	[0; 2.3]
Kumar 2019	0	84	0.0	[0; 1.6]
Lehtiranta 2020	1	308	0.3	[0; 1.0]
McNab 2014	1	338	0.3	[0; 0.9]
**Random effects model**		**915**	**0.3**	**[0; 0.7]**

Heterogeneity: $I^2 = 0\%$, $\tau^2 = 0$, $\chi^2_5 = 0.32$ ($p = 1.00$)

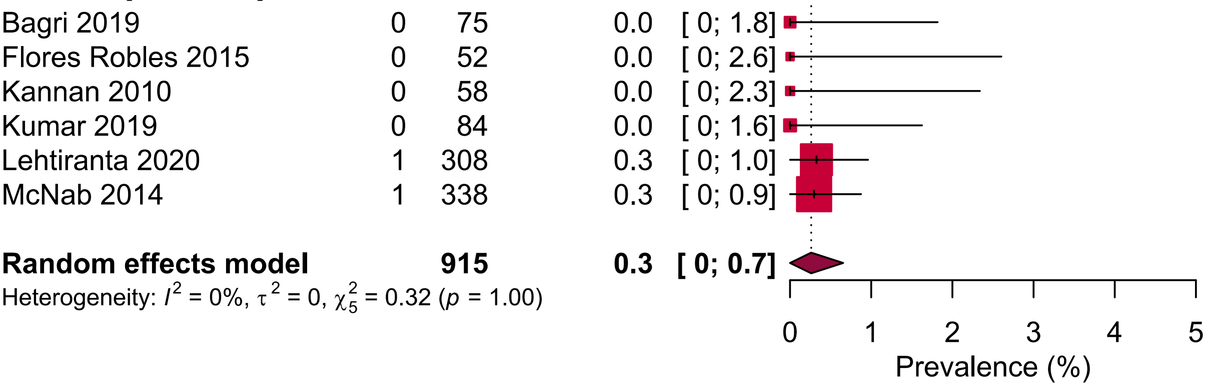

B

Study ID	Cases	Total	Prevalence	95% C.I.
**Seizure (Hypotonic)**				
Bagri 2019	0	75	0.0	[0.0; 1.8]
Flores Robles 2015	0	99	0.0	[0.0; 1.4]
Kannan 2010	1	109	0.9	[0.0; 2.7]
Kumar 2019	0	84	0.0	[0.0; 1.6]
Lehtiranta 2020	0	306	0.0	[0.0; 0.5]
McNab 2014	7	338	2.1	[0.6; 3.6]
**Random effects model**		**1011**	**0.4**	**[0.0; 1.0]**

Heterogeneity: $I^2 = 32\%$, $\tau^2 < 0.0001$, $\chi^2_5 = 7.37$ ($p = 0.19$)

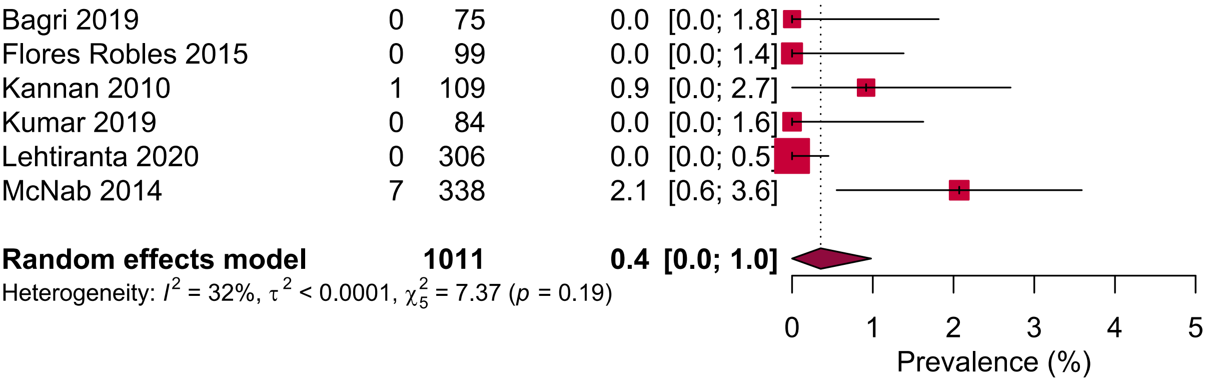

C

Study ID	Cases	Total	Prevalence	95% C.I.
**Oedema (Isotonic)**				
Bagri 2019	0	75	0.0	[0.0; 1.8]
Choong 2011	9	128	7.0	[2.6; 11.5]
Flores Robles 2015	0	52	0.0	[0.0; 2.6]
Friedman 2015	2	47	4.3	[0.0; 10.0]
Kumar 2019	0	84	0.0	[0.0; 1.6]
McNab 2014	18	338	5.3	[2.9; 7.7]
**Random effects model**		**724**	**2.3**	**[0.0; 4.6]**

Heterogeneity: $I^2 = 79\%$, $\tau^2 = 0.0006$, $\chi^2_5 = 23.68$ ($p < 0.01$)

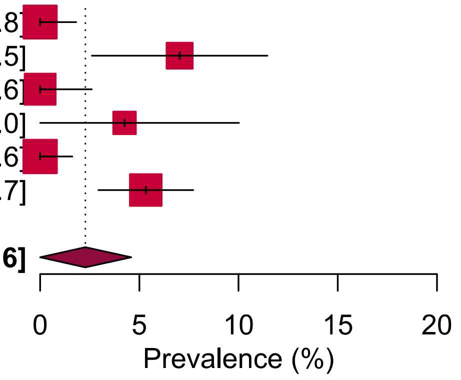

D

Study ID	Cases	Total	Prevalence	95% C.I.
**Oedema (Hypotonic)**				
Bagri 2019	0	75	0.0	[0.0; 1.8]
Choong 2011	8	130	6.2	[2.0; 10.3]
Flores Robles 2015	0	99	0.0	[0.0; 1.4]
Friedman 2015	0	45	0.0	[0.0; 3.0]
Kumar 2019	0	84	0.0	[0.0; 1.6]
McNab 2014	12	338	3.6	[1.6; 5.5]
**Random effects model**		**771**	**1.2**	**[0.0; 2.8]**

Heterogeneity: $I^2 = 71\%$, $\tau^2 = 0.0002$, $\chi^2_5 = 17.15$ ($p < 0.01$)

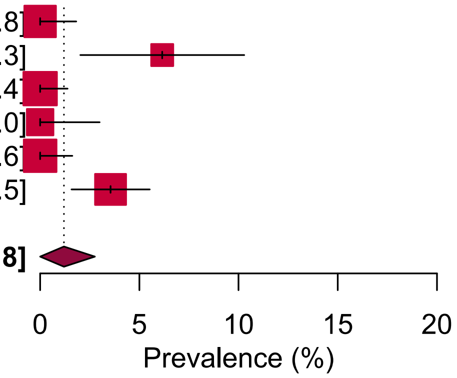

E

Study ID	Cases	Total	Prevalence	95% C.I.
**Hypertension (Isotonic)**				
Choong 2011	0	128	0.0	[0.0; 1.1]
Flores Robles 2015	0	52	0.0	[0.0; 2.6]
Friedman 2015	2	47	4.3	[0.0; 10.0]
Montanana 2008	7	59	11.9	[3.6; 20.1]
**Random effects model**		**286**	**1.9**	**[0.0; 4.9]**

Heterogeneity: $I^2 = 69\%$, $\tau^2 = 0.0005$, $\chi^2_3 = 9.70$ ($p = 0.02$)

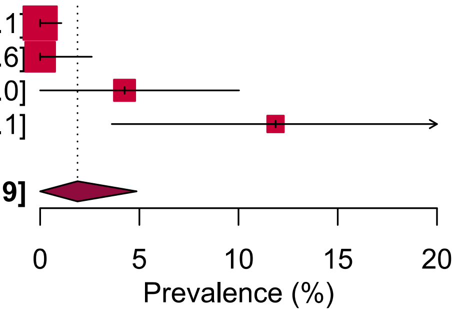

F

Study ID	Cases	Total	Prevalence	95% C.I.
**Hypertension (Hypotonic)**				
Choong 2011	2	130	1.5	[0.0; 3.7]
Flores Robles 2015	0	99	0.0	[0.0; 1.4]
Friedman 2015	2	45	4.4	[0.0; 10.5]
Montanana 2008	7	63	11.1	[3.4; 18.9]
**Random effects model**		**337**	**2.3**	**[0.0; 5.2]**

Heterogeneity: $I^2 = 70\%$, $\tau^2 = 0.0005$, $\chi^2_3 = 9.94$ ($p = 0.02$)

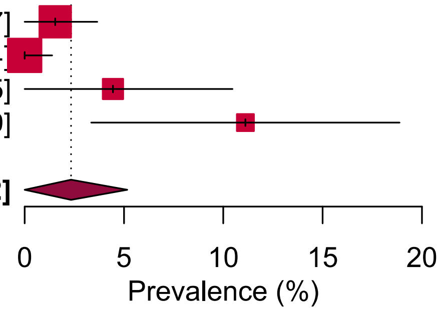

G

Study ID	Cases	Total	Prevalence	95% C.I.
**Metabolic Acidosis (Isotonic)**				
Torres 2019	38	145	26.2	[19; 33.4]

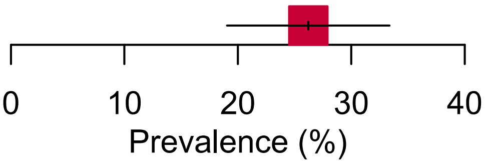

H

Study ID	Cases	Total	Prevalence	95% C.I.
**Metabolic Acidosis (Hypotonic)**				
Torres 2019	32	154	20.8	[14.4; 27.2]

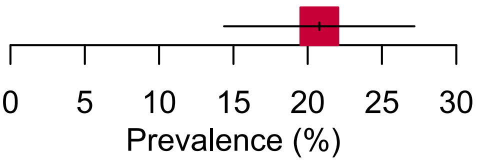

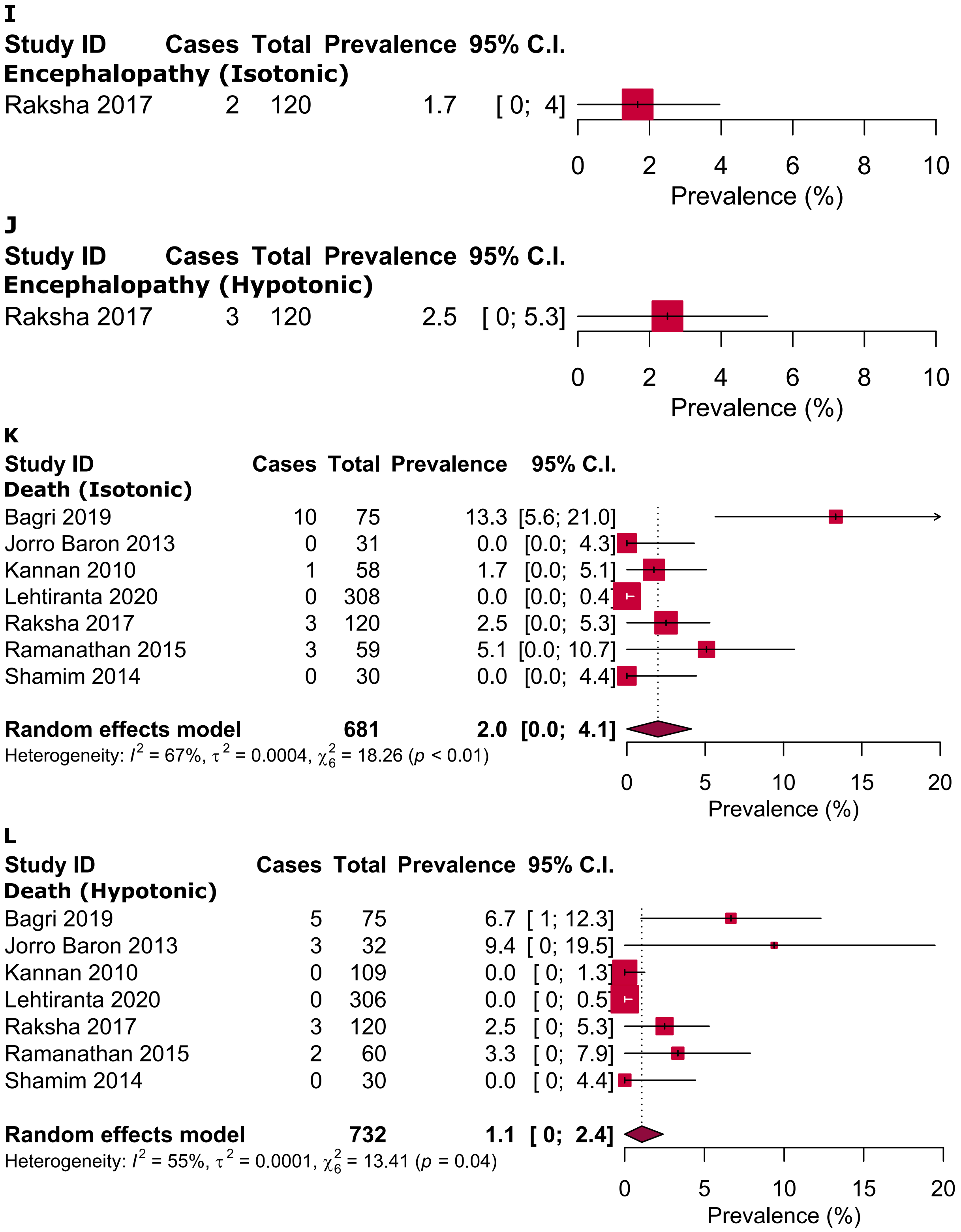

Figure S5. Prevalence of adverse events following isotonic and hypotonic fluids in hospitalised children.
