# Supplementary material for: Efficacy and Safety of Isotonic and Hypotonic Intravenous Maintenance Fluids in Hospitalised Children: A Systematic Review and Meta-Analysis of Randomised Controlled Trials
Source: Children (Basel). 2021 Sep 8;8(9):785. doi: 10.3390/children8090785 (PMC8471545; doi:10.3390/children8090785)
Supplement: Supplementary file 1 [file children-08-00785-s001.zip › Figure S6_Adverse events_RevMan.pdf]

(A) Seizure

Study or Subgroup	Isotonic Events	Isotonic Total	Hypotonic Events	Hypotonic Total	Weight	Risk Ratio M-H, Random, 95% CI
Kumar 2019	0	84	0	84		Not estimable
Bagri 2019	0	75	0	75		Not estimable
Flores Robles 2015	0	52	0	99		Not estimable
McNab 2014	1	338	7	338	48.7%	0.14 [0.02, 1.15]
Kannan 2010	0	58	1	109	25.7%	0.62 [0.03, 15.02]
Lehtiranta 2020	1	308	0	306	25.6%	2.98 [0.12, 72.88]
**Total (95% CI)**		**915**		**1011**	**100.0%**	**0.45 [0.08, 2.67]**
Total events	2		8			

Heterogeneity: Tau² = 0.54; Chi² = 2.54, df = 2 (P = 0.28); I² = 21%
Test for overall effect: Z = 0.87 (P = 0.38)

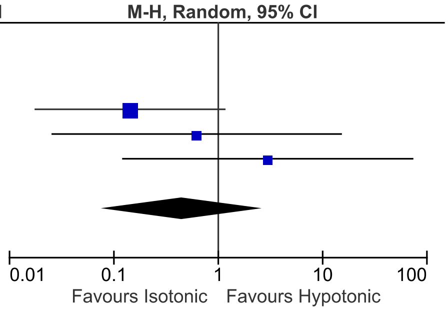

(B) Oedema

Study or Subgroup	Isotonic Events	Isotonic Total	Hypotonic Events	Hypotonic Total	Weight	Risk Ratio M-H, Random, 95% CI
Bagri 2019	0	75	0	75		Not estimable
Flores Robles 2015	0	52	0	99		Not estimable
Kumar 2019	0	84	0	84		Not estimable
Choong 2011	9	128	8	130	36.3%	1.14 [0.46, 2.87]
McNab 2014	18	338	12	338	60.3%	1.50 [0.73, 3.07]
Friedman 2015	2	47	0	45	3.4%	4.79 [0.24, 97.14]
**Total (95% CI)**		**724**		**771**	**100.0%**	**1.41 [0.81, 2.46]**
Total events	29		20			

Heterogeneity: Tau² = 0.00; Chi² = 0.87, df = 2 (P = 0.65); I² = 0%
Test for overall effect: Z = 1.22 (P = 0.22)

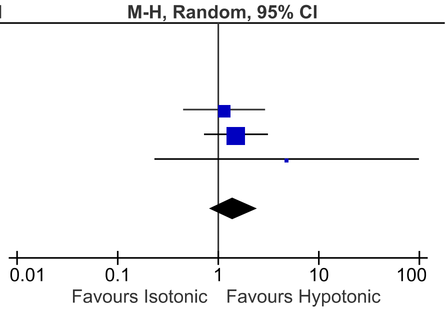

(C) Hypertension

Study or Subgroup	Isotonic Events	Isotonic Total	Hypotonic Events	Hypotonic Total	Weight	Risk Ratio M-H, Random, 95% CI
Saba 2011	0	16	0	21		Not estimable
Flores Robles 2015	0	52	0	99		Not estimable
Choong 2011	0	128	2	130	6.5%	0.20 [0.01, 4.19]
Friedman 2015	2	47	2	45	16.1%	0.96 [0.14, 6.51]
Montanana 2008	7	23	7	23	77.5%	1.00 [0.42, 2.40]
**Total (95% CI)**		**266**		**318**	**100.0%**	**0.90 [0.42, 1.93]**
Total events	9		11			

Heterogeneity: Tau² = 0.00; Chi² = 1.04, df = 2 (P = 0.59); I² = 0%
Test for overall effect: Z = 0.28 (P = 0.78)

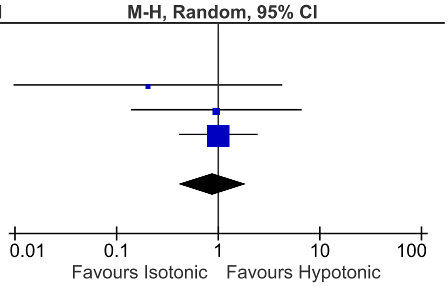

(D) Metabolic acidosis

Study or Subgroup	Isotonic Events	Isotonic Total	Hypotonic Events	Hypotonic Total	Weight	Risk Ratio M-H, Random, 95% CI
Torres 2019	38	145	32	154	100.0%	1.26 [0.84, 1.90]
**Total (95% CI)**		**145**		**154**	**100.0%**	**1.26 [0.84, 1.90]**
Total events	38		32			

Heterogeneity: Not applicable
Test for overall effect: Z = 1.10 (P = 0.27)

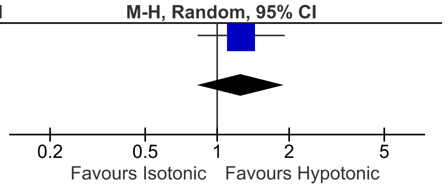

(E) Encephalopathy

Study or Subgroup	Isotonic Events	Isotonic Total	Hypotonic Events	Hypotonic Total	Weight	Risk Ratio M-H, Random, 95% CI
Raksha 2017	2	120	3	120	100.0%	0.67 [0.11, 3.92]
**Total (95% CI)**		**120**		**120**	**100.0%**	**0.67 [0.11, 3.92]**
Total events	2		3			

Heterogeneity: Not applicable
Test for overall effect: Z = 0.45 (P = 0.65)

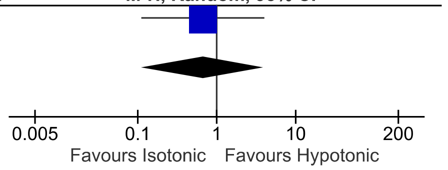

(F) Death

Study or Subgroup	Isotonic Events	Isotonic Total	Hypotonic Events	Hypotonic Total	Weight	Risk Ratio M-H, Random, 95% CI
Lehtiranta 2020	0	308	0	306		Not estimable
Shamim 2014	0	30	0	30		Not estimable
Jorro Baron 2013	0	31	3	32	6.2%	0.15 [0.01, 2.74]
Raksha 2017	3	120	3	120	21.1%	1.00 [0.21, 4.86]
Ramanathan 2015	3	59	2	60	17.2%	1.53 [0.26, 8.80]
Bagri 2019	10	75	5	75	50.3%	2.00 [0.72, 5.57]
Kannan 2010	1	58	0	109	5.2%	5.59 [0.23, 135.17]
**Total (95% CI)**		**681**		**732**	**100.0%**	**1.48 [0.72, 3.06]**
Total events	17		13			

Heterogeneity: Tau² = 0.00; Chi² = 3.70, df = 4 (P = 0.45); I² = 0%
Test for overall effect: Z = 1.06 (P = 0.29)

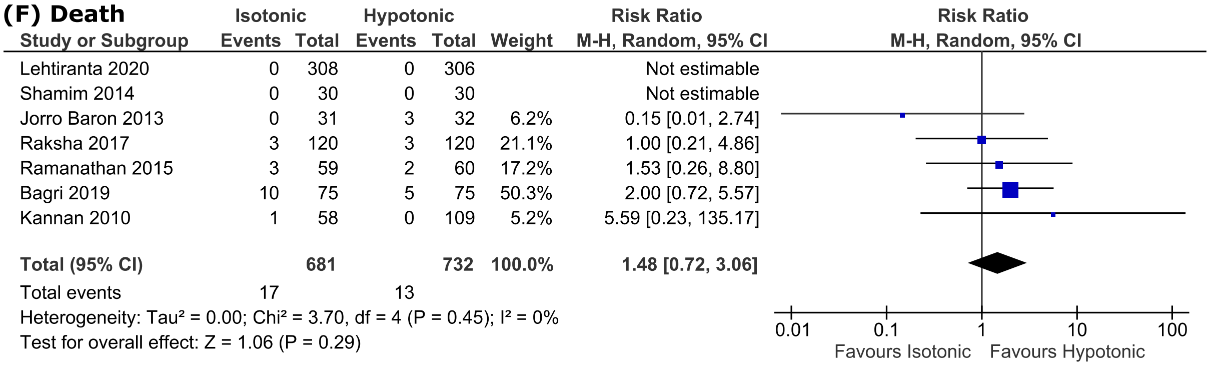

Figure S6. Risk of adverse events following isotonic and hypotonic fluids in hospitalised children.
