# Supplementary material for: Efficacy and Safety of Isotonic and Hypotonic Intravenous Maintenance Fluids in Hospitalised Children: A Systematic Review and Meta-Analysis of Randomised Controlled Trials
Source: Children (Basel). 2021 Sep 8;8(9):785. doi: 10.3390/children8090785 (PMC8471545; doi:10.3390/children8090785)
Supplement: Supplementary file 1 [file children-08-00785-s001.zip › Figure S7_Publication Bias.pdf]

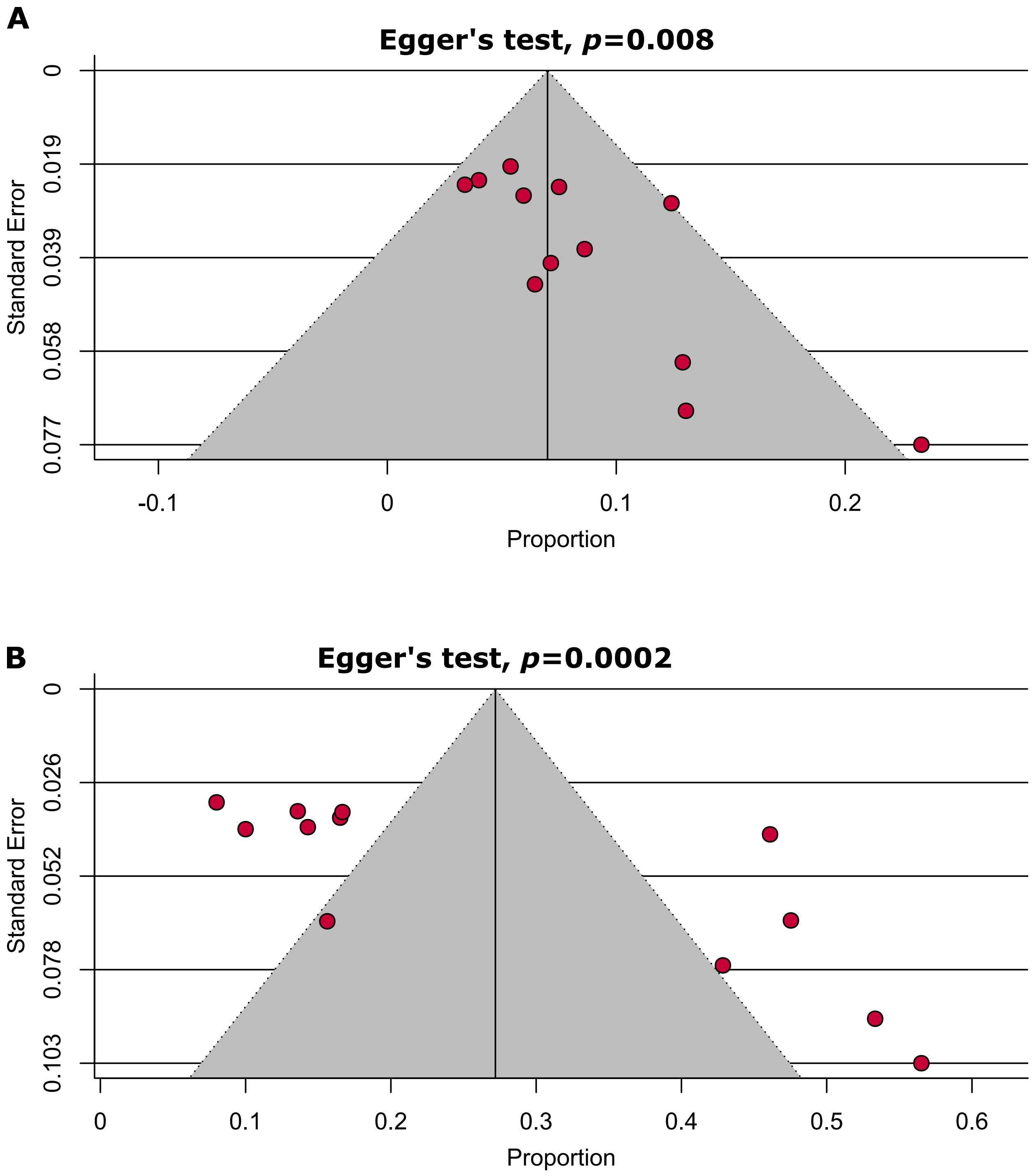

A
Egger's test, p=0.008
Standard Error
0
0.019
0.039
0.058
0.077
-0.1
0
0.1
0.2
Proportion
B
Egger's test, p=0.0002
Standard Error
0
0.026
0.052
0.078
0.103
0
0.1
0.2
0.3
0.4
0.5
0.6
Proportion

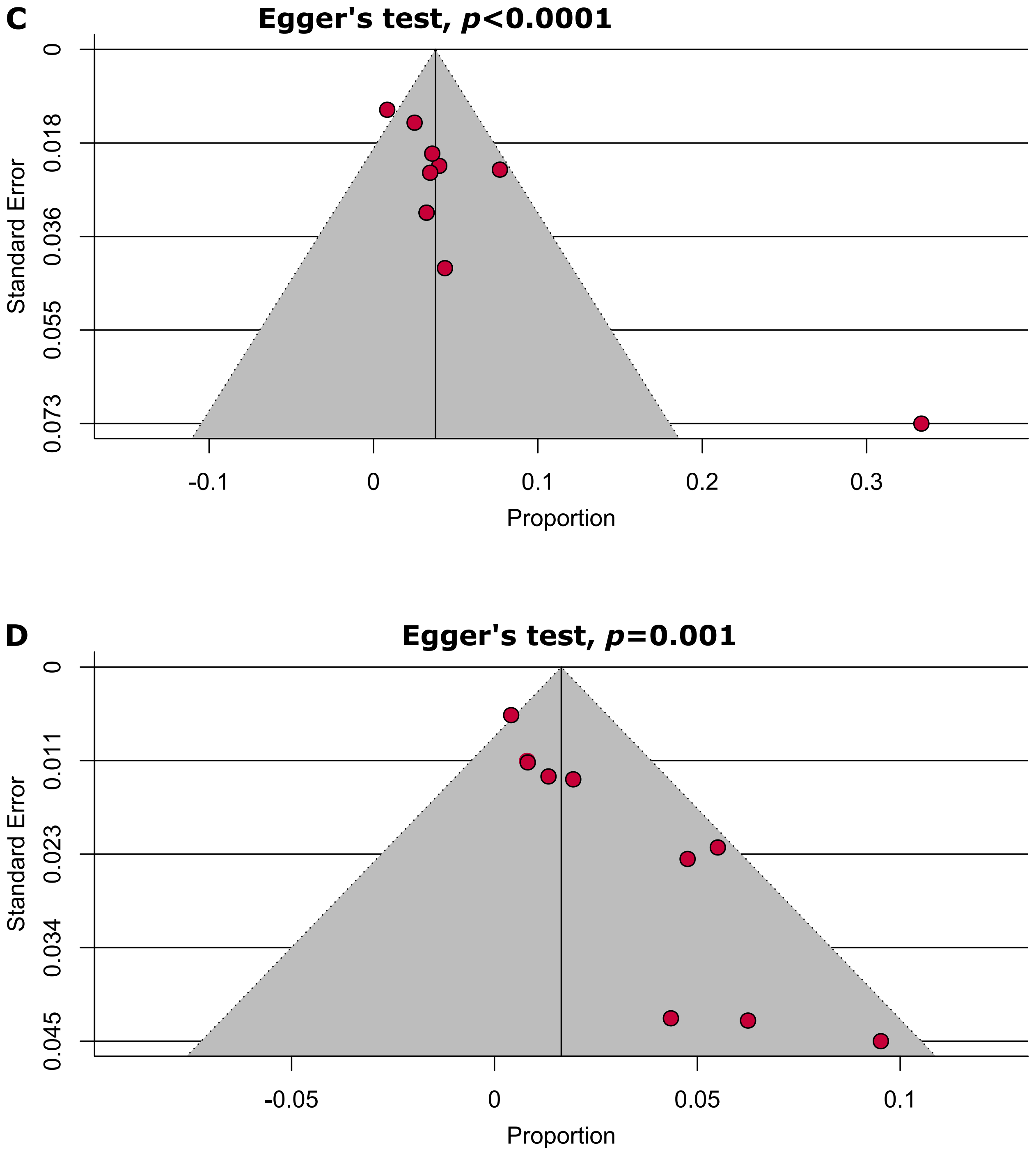

Figure S7. Funnel plots representing significant publication bias in estimating the prevalence of hyponatraemia followed by (A) Isotonic and (B) hypotonic fluids and hypernatraemia followed by (C) Isotonic and (D) hypotonic fluids.
