# Supplementary material for: Efficacy and Safety of Isotonic and Hypotonic Intravenous Maintenance Fluids in Hospitalised Children: A Systematic Review and Meta-Analysis of Randomised Controlled Trials
Source: Children (Basel). 2021 Sep 8;8(9):785. doi: 10.3390/children8090785 (PMC8471545; doi:10.3390/children8090785)
Supplement: Supplementary file 1 [file children-08-00785-s001.zip › Table S1_Search Strategy.pdf]

Table S1. Database search strategies

Databases	Search strategies
**PubMed**	((isotonic[Title/Abstract] OR hypotonic[Title/Abstract] OR saline[Title/Abstract]) AND (hyponatremia[Title/Abstract] OR hyponatraemia[Title/Abstract])) AND (children[Title/Abstract] OR pediatric[Title/Abstract] OR paediatric[Title/Abstract] OR pediatrics[Title/Abstract] OR paediatrics[Title/Abstract] OR adolescent[Title/Abstract] OR adolescents[Title/Abstract] OR child[Title/Abstract] OR infant[Title/Abstract] OR infants[Title/Abstract] OR newborn[Title/Abstract] OR newborns[Title/Abstract])
**Web of Science**	TI=(isotonic OR hypotonic) AND TI=(children OR pediatric OR paediatric OR pediatrics OR paediatrics OR adolescent OR adolescents OR child OR infant OR infants OR newborn OR newborns)
**Scopus**	TITLE-ABS(isotonic OR hypotonic OR saline) AND TITLE-ABS(hyponatremia OR hyponatraemia) AND TITLE-ABS(children OR pediatric OR paediatric OR pediatrics OR paediatrics OR adolescent OR adolescents OR child OR infant OR infants OR newborn OR newborns)
**Google Scholar**	allintitle: (isotonic OR hypotonic) (children OR pediatric OR paediatric OR pediatrics OR paediatrics OR adolescent OR adolescents OR child OR infant OR infants OR newborn OR newborns)
**Cochrane Library**	((isotonic OR hypotonic) AND (children OR pediatric OR paediatric OR pediatrics OR paediatrics OR adolescent OR adolescents OR child OR infant OR infants OR newborn OR newborns)):ti
