# Supplementary material for: Efficacy and Safety of Isotonic and Hypotonic Intravenous Maintenance Fluids in Hospitalised Children: A Systematic Review and Meta-Analysis of Randomised Controlled Trials
Source: Children (Basel). 2021 Sep 8;8(9):785. doi: 10.3390/children8090785 (PMC8471545; doi:10.3390/children8090785)
Supplement: Supplementary file 1 [file children-08-00785-s001.zip › Table S2_Sensitivity.pdf]

Table S2. Sensitivity analyses

Strategies of Sensitivity analyses	Risk Ratio or Mean Difference [95% CIs] (%)	Number of studies analysed	Difference of results	Total number of subjects	Heterogeneity	
					I^2	*p*-value
**Hyponatremia at 24 hours**						
Excluding small studies (n<100)	0.36 [0.26-0.48]	7	0.01 higher	1376	0%	0.83
Excluding low- or medium-quality studies	0.33 [0.25-0.44]	10	0.02 lower	1248	0%	0.48
Using a fixed-effects model	0.33 [0.26-0.42]	12	0.02 lower	1721	0%	0.60
Excluding studies with a change of maintenance fluid	0.33 [0.25-0.44]	10	0.02 lower	1248	0%	0.48
**Hypernatraemia at 24 hours**						
Excluding small studies (n<100)	1.63 [0.66-4.05]	5	0.27 lower	1077	22%	0.27
Excluding low- or medium-quality studies	1.54 [0.77-3.07]	7	0.36 lower	889	9%	0.36
Using a fixed-effects model	2.03 [1.19-3.45]	9	0.13 higher	1362	7%	0.38
Excluding studies with a change of maintenance fluid	1.54 [0.77-3.07]	7	0.36 lower	889	9%	0.36
**Serum sodium levels at 24 hours**						
Excluding small studies (n<100)	2.40 [1.04-3.76]	6	0.13 lower	1218	89%	<0.00001
Excluding low- or medium-quality studies	2.22 [1.79-2.64]	8	0.31 lower	1006	92%	<0.00001
Using a fixed-effects model	2.14 [1.79-2.49]	10	0.39 lower	1479	90%	<0.00001
Excluding studies with a change of maintenance fluid	3.04 [1.54-4.53]	7	0.51 higher	698	86%	<0.00001
**Urine sodium levels at 24 hours**						
Excluding small studies (n<100)	50.14 [23.74-76.54]]	3	No change	1132	94%	<0.00001
Excluding low- or medium-quality studies	61.68 [53.28-70.08]	2	11.54 higher	899	0%	0.34
Using a fixed-effects model	46.08 [39.69-52.46]	3	4.06 lower	1132	94%	<0.00001

CIs: confidence intervals.
