# Supplementary material for: Efficacy and Safety of Isotonic and Hypotonic Intravenous Maintenance Fluids in Hospitalised Children: A Systematic Review and Meta-Analysis of Randomised Controlled Trials
Source: Children (Basel). 2021 Sep 8;8(9):785. doi: 10.3390/children8090785 (PMC8471545; doi:10.3390/children8090785)
Supplement: Supplementary file 1 [file children-08-00785-s001.zip › Table S3_Quality assessmnet.pdf]

Table S3. Quality assessment of the included studies.

No.	Study ID	Questions Assessing Included Studies													Yes (%)
		1	2	3	4	5	6	7	8	9	10	11	12	13	
1	Almeida 2014	Y	N	Y	N	N	N	Y	Y	Y	Y	Y	Y	Y	69.0
2	Bagri 2019	Y	Y	Y	N	N	N	Y	Y	Y	Y	Y	Y	Y	77.0
3	Balasubramaniam 2011	Y	Y	Y	Y	Y	Y	Y	Y	Y	Y	Y	Y	Y	100.0
4	Brazel 1996	U	U	Y	U	U	U	Y	Y	Y	Y	Y	Y	Y	61.0
5	Choong 2011	Y	Y	Y	Y	Y	Y	Y	Y	Y	Y	Y	Y	Y	100.0
6	Coulthard 2012	Y	Y	Y	Y	N	N	Y	Y	Y	Y	Y	Y	Y	85.0
7	Flores Robles 2015	Y	Y	Y	N	N	N	Y	Y	Y	Y	Y	Y	Y	77.0
8	Friedman 2015	Y	Y	Y	Y	Y	Y	Y	Y	Y	Y	Y	Y	Y	100.0
9	Jorro Baron 2013	Y	Y	Y	Y	Y	Y	Y	Y	Y	Y	Y	Y	Y	100.0
10	Kannan 2010	Y	Y	Y	N	N	N	Y	Y	Y	Y	Y	Y	Y	77.0
11	Kumar 2019	Y	Y	Y	N	N	N	Y	Y	Y	Y	Y	Y	Y	77.0
12	Lehtiranta 2020	Y	Y	Y	N	N	N	Y	Y	Y	Y	Y	Y	Y	77.0
13	McNab 2014	Y	Y	Y	Y	Y	Y	Y	Y	Y	Y	Y	Y	Y	100.0
14	Mierzewska-Schmidt 2015	Y	U	Y	N	N	N	Y	Y	Y	Y	Y	Y	Y	69.0
15	Montanana 2008	Y	Y	Y	U	U	U	Y	Y	Y	Y	Y	Y	Y	77.0
16	Omoifo 2018	U	U	Y	U	U	U	Y	Y	Y	Y	Y	Y	Y	61.0
17	Pemde 2015	Y	Y	Y	Y	Y	Y	Y	Y	Y	Y	Y	Y	Y	100.0
18	Ramanathan 2015	Y	Y	Y	N	N	N	Y	Y	Y	Y	Y	Y	Y	77.0
19	Raksha 2017	U	U	Y	N	N	N	Y	Y	Y	Y	Y	Y	Y	61.0
20	Saba 2011	Y	Y	Y	Y	Y	Y	Y	Y	Y	Y	Y	Y	Y	100.0
21	Shamim 2014	Y	Y	Y	N	N	N	Y	Y	Y	Y	Y	Y	Y	77.0
22	Torres 2019	Y	Y	Y	Y	Y	Y	Y	Y	Y	Y	Y	Y	Y	100.0

1. Was true randomization used for assignment of participants to treatment groups? 2. Was allocation to treatment groups concealed? 3. Were treatment groups similar at the baseline? 4. Were participants blind to treatment assignment? 5. Were those delivering treatment blind to treatment assignment? 6. Were outcomes assessors blind to treatment assignment? 7. Were treatment groups treated identically other than the intervention of interest? 8. Was follow up complete and if not, were differences between groups in terms of their follow up adequately described and analyzed? 9. Were participants analyzed in the groups to which they were randomized? 10. Were outcomes measured in the same way for treatment groups? 11. Were outcomes measured in a reliable way? 12. Was appropriate statistical analysis used? 13. Was the trial design appropriate, and any deviations from the standard RCT design (individual randomization, parallel groups) accounted for in the conduct and analysis of the trial? Y=Yes; N=No; U=Unclear or NA Not applicable
